# One-Step Reverse-Transcription Recombinase Polymerase Amplification Using Lateral Flow Strips for the Detection of Coxsackievirus A6

**DOI:** 10.3389/fmicb.2021.629533

**Published:** 2021-02-04

**Authors:** Jia Xie, Xiaohan Yang, Lei Duan, Keyi Chen, Pan Liu, Wenli Zhan, Changbin Zhang, Hongyu Zhao, Mengru Wei, Yuan Tang, Mingyong Luo

**Affiliations:** ^1^Medical Genetic Centre, Guangdong Women and Children’s Hospital, Guangzhou Medical University, Guangzhou, China; ^2^Medical Genetic Centre, Guangdong Women and Children Hospital, Guangzhou, China

**Keywords:** Coxsackievirus A6, enterovirus, recombinase polymerase amplification, lateral flow strips, rapid detection

## Abstract

Hand, foot, and mouth disease (HFMD) is a common infectious disease affecting mainly children under 5 years of age. Coxsackievirus A6 (CVA-6), a major causative pathogen of HFMD, has caused outbreaks in recent years. Currently, no effective vaccine or antiviral treatments are available. In this study, one-step reverse-transcription recombinase polymerase amplification (RT-RPA), combined with a disposable lateral flow strip (LFS) assay, was developed to detect CVA-6. This assay can be performed in less than 35 min at 37°C without expensive instruments, and the result can be observed directly with the naked eye. The sensitivity of the RT-RPA-LFS was 10 copies per reaction, which was comparable to that of the conventional real-time quantitative polymerase chain reaction (qPCR) assays. Moreover, the assay specificity was 100%. The clinical performance of the RT-RPA-LFS assay was evaluated using 142 clinical samples, and the coincidence rate between RT-RPA-LFS and qPCR was 100%. Therefore, our RT-RPA-LFS assay provides a simple and rapid approach for point-of-care CVA-6 diagnosis.

## Introduction

Hand, foot, and mouth disease (HFMD) is a common infectious disease that generally affects children under 5 years of age. It is typically characterised by fever, maculopapular rash, or blisters on the hands, feet, mouth, and buttocks (Li et al., 2018). However, a few cases may rapidly develop severe neurological and systemic complications, even leading to death. Coxsackievirus A (CVA) 2−8, 10, 12, 14, and 16, and Enterovirus A 71 (EV-A71), members of the genus *Enterovirus* within the family *Picornaviridae*, are recognised as the main etiological agents of HFMD (Solomon et al., 2010; Yang et al., 2011; Wang J. et al., 2018). Recently, numerous HFMD outbreaks caused by CVA-6 have been reported (Li et al., 2014; Sinclair et al., 2014; Anh et al., 2018; Cisterna et al., 2018; Zeng et al., 2018; Yang et al., 2020a), and the number of severe HFMD cases associated with CVA-6 is increasing (Li et al., 2020). Moreover, CVA-6-infected cases could obscure atypical clinical symptoms of HFMD, which may increase the difficulty in HFMD diagnosis and lead to the underestimation of the actual number of infected persons (Sinclair et al., 2014; Cisterna et al., 2018). Although vaccines against EV-A71 have been successfully developed, they cannot protect children from infection with CVA-6. Further, the use of these vaccines is limited to mainland China (Nguyet et al., 2020). No effective antiviral treatments have been developed for HFMD. Therefore, a simple, rapid, and sensitive detection method for CVA-6, which is crucial for the accurate treatment, prevention and control of the spread of HFMD, is needed.

The available laboratory detection methods for CVA-6 include traditional and molecular methods. The traditional method requires reference anti-sera, and reliance on virus culture is rarely applied to routine laboratory detection because of its comparatively low sensitivity, tedious operation steps, long turnaround time, and cross-immune response with other serotypes (Lin et al., 2008; Zhang et al., 2012). The most common molecular diagnostic method, real-time quantitative polymerase chain reaction (qPCR), has high sensitivity and specificity, and is the most widely used detection method for CVA-6 infections. Nevertheless, the need for expensive and large equipment and well-trained technicians may hinder the applications of qPCR in developing areas.

Recombinase polymerase amplification (RPA), a novel isothermal amplification technology, is an alternative diagnostic tool for pathogens. RPA can rapidly and specifically amplify target genes at a constant low reaction temperature, which eliminates the sophisticated thermal cyclers required for traditional molecular diagnostic methods. Over the past decade, RPA technology has dramatically progressed, and it is now used to detect many pathogens in the field, including the influenza virus, human immunodeficiency virus, and dengue virus (Boyle et al., 2013; Teoh et al., 2015; Sun et al., 2019). Currently, the RPA product can be analysed by agarose gel electrophoresis, real-time monitoring, and lateral flow strips (LFS). After the RPA, the product was labelled with FITC and biotin. By the gold nanoparticles on the LFS, it can be observed with the naked eye as colored signals (Wu et al., 2020). Compared with other detection methods, LFS are more appropriate for point-of-care testing (POCT) and one can rapidly and sensitively analyse products by visualisation.

In our previous study, we reported a simple and rapid RPA-LFS assay for enterovirus (Yang et al., 2020b). To further improve the practicability of RPA, we developed and evaluated a one-step reverse transcription recombinase polymerase amplification (RT-RPA) combined with a disposable LFS device to detect CVA-6, which will be an attractive diagnostic tool for POCT.

## Materials and Methods

### Clinical Sample Collection and Virus Isolation

A total of 142 stool samples were collected from children with suspected HFMD who were admitted to Guangdong Women and Children’s Hospital (Guangzhou, Guangdong, China). Total RNA was extracted using EX-DNA/RNA Virus Kits (Suzhou Tianlong Bio-technology Co., Ltd., Suzhou, China). These samples were tested in parallel using RT-RPA-LFS and qPCR to investigate the clinical performance of the RT-RPA-LFS assay versus the standard qPCR. A panel of nucleic acids from other pathogens, including non-CVA-6 enteroviruses [CVA-2, 4, 5, 9, 10, 16, EV-A71, CVB-2, 3, and Echovirus (E) 3, 11, 18] and pathogens infecting similar areas in the body (adenovirus, rotavirus, and norovirus), served as controls to evaluate assay specificity. All pathogens were isolated from clinical samples and stored at −80°C in the laboratory.

This study was approved by the ethics committee of Guangdong Women and Children’s Hospital. All information collected from patients was anonymised prior to analysis, and informed consent was waived.

### Primer and Probe Design

The VP1 gene, the domain of high genetic variability, is widely used to identify enteroviruses (Oberste et al., 2006). Due to genetic recombination and enterovirus variability, the CVA-6 reference sequences submitted to the GenBank database in the last 5 years were downloaded and all sequences aligned to identify highly conserved regions of the VP1 gene (Supplementary Material 1). RPA primers and probe were designed to target the highly conserved regions of VP1, according to the TwistAmp nfo kit, following the manufacturer’s guidelines. Primer and probe specificity were verified by BLAST analysis on the NCBI database^1^. Verified primers and probe were synthesised by Sangon Biotech (Shanghai, China). Detailed information on the primers and probe is listed in Table 1.

**TABLE 1:** Primer and probe sequences used for RT-RPA-LFS.

Primer/Probe	Sequence (5′-3′)	Genomic position	Product size (bp)
RT-RPA-F	[Biotin]GGAGTTGTAGAGGTRAAGGACTCGGGYACTA	259–289	–
RT-RPA-R	TCCGGCTTAGGGGCYCCTGGTGGYACATACAT	445–476	218
RT-RPA-P	[FAM]AGGTTGGACACAAAAGTGAACTCRGCATCAA[dSpacer]GCGCATGTATGTTGA[C3-spacer]	361–407	149

*FAM: Carboxyfluorescein; dSpacer: A tetrahydrofuran residue; C3-spacer, 3′-block.*

### Generation of the RNA Standard

A 1110-bp fragment covering the entire CVA-6 VP1 gene was amplified as described previously (Tan et al., 2015). The products were purified and cloned into the pGEM-T vector. Then, the plasmids were linearized, transcribed using T7 RNA polymerase, and digested with DNsae I. The RNA standard was purified by phenol-chloroform extraction. The RNA concentration was quantified using the Nanodrop 2000 spectrophotometer (Thermo Scientific, United States), and the RNA copy number was calculated using the following formula: RNA copy number (copy/μL) = [RNA concentration (g/μL) × 10^–9^]/(RNA in length × 340) × 6.02 × 10^23^. The RNA standard was stored at −80°C prior to use.

### RT-RPA-LFS Assay

The RT-RPA assay was performed in a volume of 50 μL using TwistAmp nfo kits (TwistDx, Cambridge, England), following the manufacturer’s protocol. MMLV reverse transcriptase (Takara, Dalian, China) was added into the reaction system. Briefly, the reaction mixture contained 29.5 μL of rehydration buffer, 2.1 μL of each primer (10 μM), 0.6 μL of probe (10 μM), 0.5 μL of reverse transcriptase (200 U/μL), 3 μL of RNA template, and 9.7 μL of nuclease-free water. Then, 2.5 μL of magnesium acetate solution (280 mM) was added to initiate the reaction. After brief mixing and centrifugation, the reaction was incubated at 37°C for 35 min in a heat block. The RPA products were detected via LFS (Milenia Biotec GmbH, Germany), and the results were observed directly with the naked eye. In addition, a new disposable lateral flow cassette (Ustar, Hangzhou, China) was used to analyse the results. The samples were considered positive when red lines appeared simultaneously at the test and control lines, while a negative result constituted only one red line at the control line.

### RT-RPA-LFS Sensitivity and Specificity

To determine the sensitivity of the RT-RPA-LFS assay, ten-fold serial dilutions of a CVA-6 RNA standard ranging from 5 × 10^6^ to 5 × 10^0^ copies/μL were used as reaction templates. Additionally, a series of CVA-6 RNA standards from 5 × 10^6^ to 5 × 10^0^ copies/μL were spiked into stool samples to assess the assay sensitivity, which can reflect the actual clinical performance. For comparison, all the samples were also tested using a commercial qPCR diagnostic kit (Suzhou Tianlong Bio-technology Co., Ltd., Suzhou, China). One microliter of each dilution was used as a template to determine the sensitivity. Finally, the RT-RPA assay specificity was assessed using non-CVA-6 enteroviruses (CVA-2, 4, 5, 9, 10, 16, EV-A71, CVB-2, 3, and E-3, 11, 18), and five samples of each enterovirus were selected in 5 independent tests. In addition, the RT-RPA assay specificity was also determined by pathogens infecting similar areas in the body (adenovirus, rotavirus, and norovirus). All assays were performed in triplicate.

### Validation of the RT-RPA-LFS Assay Using Clinical Samples

To validate the clinical performance of the RT-RPA-LFS assay, 142 clinical samples collected from children with suspected HFMD were analysed using a commercial qPCR diagnostic kit in parallel with the RT-RPA-LFS assay.

## Results

### Optimisation of the RT-RPA-LFS Assay

**FIGURE 1:**
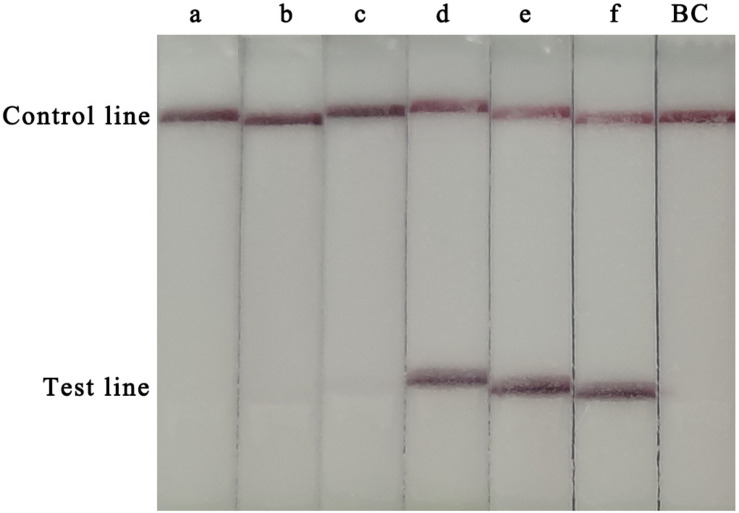
Optimization of the RT-RPA-LFS assay temperature. Temperatures from 20–42°C were tested to determine the optimal reaction temperature of the RT-RPA-LFS assay. a, 20°C; b, 30°C; b, 35°C; d, 37°C; e, 40°C; f, 42°C; BC, blank control.

**FIGURE 2:**
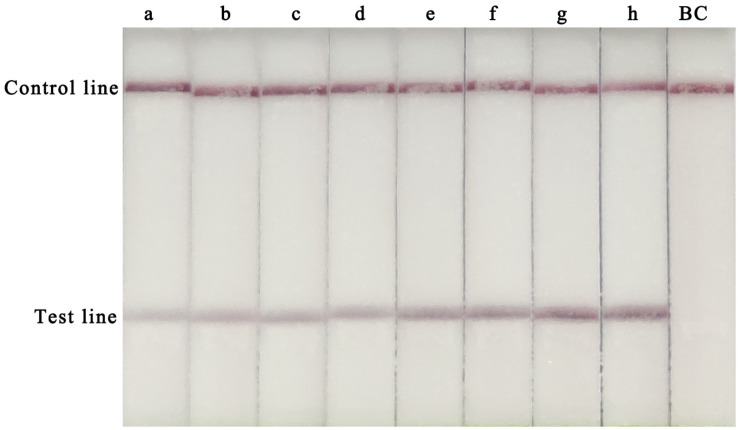
Optimization of the RT-RPA-LFS assay reaction time. Reaction times from 5 to 40 min were tested to determine the optimal reaction time of the RT-RPA-LFS assay. a, 5 min; a, 10 min; c, 15 min; d, 20 min; e, 25 min; f, 30 min; g, 35 min; h, 40 min; BC, blank control.

To determine the optimal assay conditions, two main RPA parameters, time and temperature, were optimised. For this experiment, 10^5^ copies of the CVA-6 RNA standard were used as the template. Several temperatures (20, 30, 35, 37, 40, and 42°C) and times (5, 10, 15, 20, 25, 30, 35, and 40 min) were tested to determine the optimal reaction conditions. A red line was observed in the test lines and control lines for reactions carried out at 37–42°C, with no clear differences observed at these temperatures (Figure 1). Hence, a conventional temperature of 37°C was considered optimal for the RT-RPA-LFS assay. The template was successfully amplified under different reaction times, and the brightest test lines appeared in the reactions that were run for 35 and 40 min (Figure 2). Thus, 35 min was chosen as the optimal reaction time and used in subsequent experiments.

### Sensitivity and Specificity of the RT-RPA-LFS Assay

**FIGURE 3:**
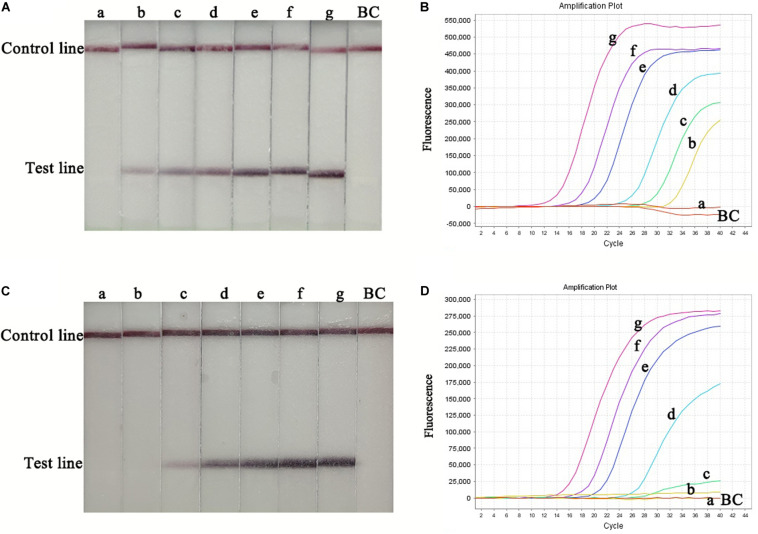
Sensitivity of the RT-RPA-LFS and qPCR assays. Serially diluted RNA standards were used to determine the sensitivity of the RT-RPA-LFS **(A)** and qPCR **(B)** assays. Serially diluted spiked-in samples were used to determine the sensitivity of the RT-RPA-LFS **(C)** and qPCR **(D)** assays. a, 1.0 × 10^0^ copies; b, 1.0 × 10^1^ copies; c, 1.0 × 10^2^ copies; d, 1.0 × 10^3^ copies; e, 1.0 × 10^4^ copies; f, 1.0 × 10^5^ copies; g, 1.0 × 10^6^ copies; BC, blank control.

**FIGURE 4:**
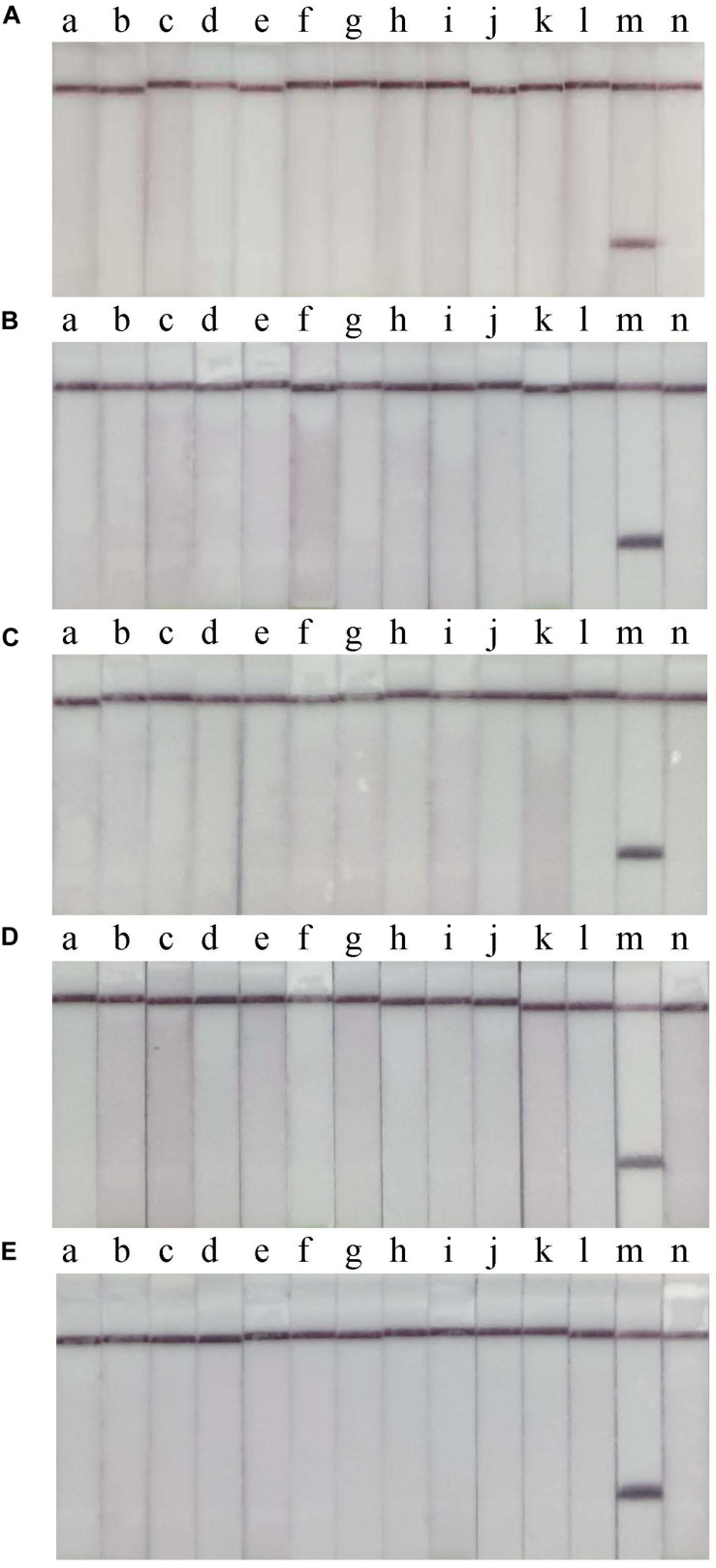
Specificity of the RT-RPA-LFD assay. Non-CVA-6 enteroviruses were used to determine the specificity of the RT-RPA-LFS assay in 5 independent tests **(A–E)**. a, CVA-2; b, CVA-4; c, CVA-5; d, CVA-9; e, CVA-10; f, CVA-16; g, EV-A71; h, CVB-2; i, CVB-3; j, E-3; k, E-11; l, E18; m, positive control; n, blank control.

RT-RPA-LFS assay sensitivity was determined using ten-fold serial dilutions of CVA-6 RNA standard and spiked samples from 10^6^ to 10^0^ copies/μL. The detection limit of the RT-RPA-LFS assay was 10^1^ and 10^2^ copies/reaction for RNA standard and spiked samples, respectively (Figure 3). These results were similar to the qPCR results. Next, a panel of nucleic acids from other pathogens were tested to evaluate the RT-RPA-LFS assay specificity. Only CVA-6 generated positive signals, and non-specific amplification was not observed with other pathogens, including non-CVA-6 enteroviruses (Figure 4) or pathogens infecting similar areas in the body (results not shown). Sensitivity and specificity results were consistent across three replicates. The results indicate that the RT-RPA-LFS assay developed in this study has high sensitivity and specificity for CVA-6 detection.

### Validation of the RT-RPA-LFS Assay Using Clinical Samples

**FIGURE 5:**
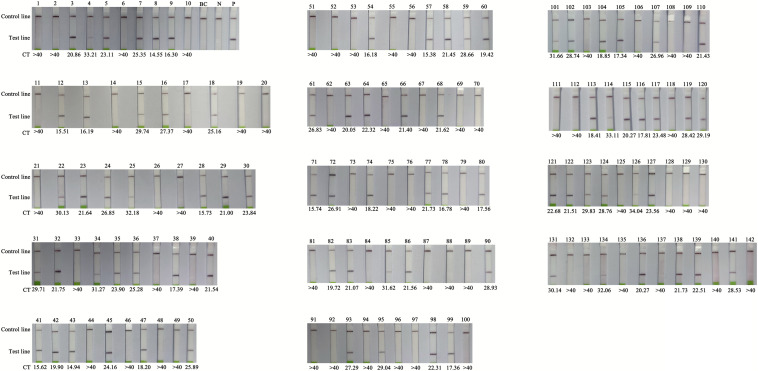
Comparison of RT-RPA-LFS with q-PCR performed on clinical samples. BC, blank control; N, negative control; P, positive control; CT, Cycle threshold, CT > 40 as negative.

The clinical performance of the RT-RPA assay was assessed by testing 142 clinical samples from children with suspected HFMD. All the samples were analysed using a commercial qPCR diagnostic kit and the RT-RPA-LFS assay. There were 81 positive and 61 negative samples identified by the RT-RPA-LFS assay, consistent with the qPCR results (Table 2 and Figure 5). Therefore, with q-PCR as the reference assay, the diagnostic sensitivity and specificity of the RT-RPA-LFS assay were 100%. In addition, a disposable lateral flow cassette was used to analyse the samples in this study (Supplementary Material 2). The device can successfully detect CVA-6 and eliminate potential contamination in a sealed room. These results demonstrate that the RT-PRA-LFS assay readily detects CVA-6 in clinical samples, similar to qPCR.

**TABLE 2:** Diagnostic performance comparison between RT-RPA-LFS and qPCR assays in clinical samples.

		qPCR		Total
		
		Positive	Negative	
RT-RPA-LFS	Positive	81	0	81
	Negative	0	61	61
	Total	81	61	142

## Discussion

HFMD, a common infectious disease, is a severe public health problem that affects millions of young children each year in China (Xing et al., 2014). CVA-6 has replaced EV-A71 and CVA-16 as the main pathogens responsible for HFMD outbreaks in recent years (Bian et al., 2015). Importantly, atypical clinical symptoms of CVA-6 infection may cause delays in diagnosis, thereby allowing HFMD to spread. Therefore, there is an urgent need to develop a simple, rapid, and accurate detection method for the early diagnosis of CVA-6 infection to improve clinical outcomes and prevent transmission. Currently, highly specific and sensitive molecular methods are the most common diagnostic methods for CVA-6. However, these methods require well-equipped laboratories and skilled personnel, making on-site detection infeasible. A novel isothermal amplification technology called the one-step RT-RPA-LFS method was developed in this study to compensate for the above shortcomings. Our method may be an ideal detection tool in resource-poor settings.

The RT-RPA-LFS method uses reverse-transcription combined with RPA to amplify the target sequence in one tube. The results are generated using a disposable LFS device in less than 35 min at 37°C without any additional steps. The moderate reaction temperature of the RT-RPA-LFS assay shows that the assay can be incubated using a simple heating block, or even body temperature, which makes on-site detection possible. In the specificity analysis, the RT-RPA-LFS assay accurately and specifically detected CVA-6, with no cross-reaction with other enteroviruses used in the study, which indicated that the design of primers and probe was important for the specificity of RPA. In addition, the detection limit of the assay was 10 copies/reaction, which was comparable with that of qPCR. Furthermore, RT-RPA-LFS and commercial q-PCR were performed in parallel using clinical samples to evaluate clinical performance, and the results were 100% consistent. Moreover, the visualised results using LFS can easily be read without any training. Together, our results indicate that the RT-RPA-LFS assay is an alternative for on-site, rapid, and primary CVA-6 detection.

In recent years, RPA combined with visual LFS has been widely developed and employed to detect pathogens (Liu et al., 2018; Sun et al., 2019; Gao et al., 2020; Yang et al., 2020b). To detect RNA viruses, an additional reverse transcription step is required before RPA-LFS. To the best of our knowledge, reverse transcription and RPA were performed in different tubes in most previous reports (Sun et al., 2019; Gao et al., 2020; Yang et al., 2020b). However, some studies attempted to integrate reverse transcription with RPA (Liu et al., 2018; Sun et al., 2018). A one-step RT-RPA assay was developed by Liu et al. (2018), to detect foot-and-mouth disease virus, and the performance of this assay was comparable to that of qPCR (Liu et al., 2018). We developed a one-step RT-RPA assay to detect CVA-6. This assay integrates reverse transcription with RPA in a single tube, which can simplify the process, shorten the operation time, and prevent contamination. Therefore, the one-step RT-RPA assay is a promising tool for field diagnostics. Potential amplicon contamination is a major drawback of RPA, which may produce false-positive results, especially in small and primary laboratories. Currently, some measures have been developed to address this problem. dUTP and Uracil-N-glycosylase (UNG) can be added to the amplification, which effectively prevents false-positive results caused by amplicon contamination. These reagents have been successfully integrated with loop-mediated isothermal amplification (Wang Y. et al., 2018). The UNG enzyme hydrolyses any dUTP-containing product before amplification, which can eliminate amplicons from previous experiments. A disposable, enclosed device was another measure that could be used to prevent potential amplicon contamination (Sun et al., 2019). In this study, a commercial disposable LFS device was employed to detect the product in a sealed chamber. Thus, our one-step RT-RPA assay combined with a disposable LFS device can improve the practicability of RPA, which will be an ideal diagnostic tool for the detection of CVA-6 in the field and for POCT.

There were two limitations in the current study. First, only stool samples were tested to evaluate the clinical performance of RT-RPA-LFS. Different types of clinical samples, including blood, cerebrospinal fluid, and vesicular fluid, should be collected to evaluate the RT-RPA-LFS assay more comprehensively in further investigations. Second, sample preparation, amplification, and analysis were conducted separately in the RT-RPA-LFS assay. Recently, microfluidic handling systems that integrate all the detection processes have been developed (Park et al., 2017; Gorgannezhad et al., 2019; Dong et al., 2021). Compared with traditional macroscale counterparts, these systems have numerous unmatchable advantages, such as automation, portability, fast analysis time, small volume consumption, and high throughput (Park et al., 2017; Gorgannezhad et al., 2019). Thus, fully automated microfluidic devices combined with RPA are strong candidates for POCT, which can achieve sample-in-answer-out for nucleic acid analysis.

In conclusion, we developed a highly sensitive and specific one-step RT-RPA-LFS assay that can easily, rapidly, and visually detect CVA-6 without any expensive instruments. This assay has great potential for becoming an ideal diagnostic tool for the rapid and accurate detection of CVA-6 infection in resource-limited areas.

## Data Availability Statement

The raw data supporting the conclusions of this article will be made available by the authors, without undue reservation.

## Author Contributions

JX, XY, and ML designed the experiments and wrote the manuscript. JX, LD, CZ, PL, HZ, MW, and YT performed the experiments. KC and WZ analyzed the data. All authors contributed to the article and approved the submitted version.

## Conflict of Interest

The authors declare that the research was conducted in the absence of any commercial or financial relationships that could be construed as a potential conflict of interest.

## Footnotes

1 http://www.ncbi.nlm.nih.gov/tools/primer-blast/

## Supplementary Material

Supplementary Material 1 The details of the VP1 gene sequences used for primer and probe design in the RT-RPA-LFS assay.

Supplementary Material 2 Detection of CVA-6 using the enclosed disposable device. P, positive control; N, negative control.

## Abbreviations

HFMD: hand foot and mouth disease
CV: Coxsackievirus
EV-A: enterovirus A
qPCR: real-time quantitative polymerase chain reaction
RPA: recombinase polymerase amplification
LFS: lateral flow strips
POCT: point-of-care testing
E: echovirus.
