# Supplementary material for: One-Step Reverse-Transcription Recombinase Polymerase Amplification Using Lateral Flow Strips for the Detection of Coxsackievirus A6
Source: Front Microbiol. 2021 Feb 4;12:629533. doi: 10.3389/fmicb.2021.629533 (PMC7889601; doi:10.3389/fmicb.2021.629533)
Supplement: Supplementary Material 1 — The details of the VP1 gene sequences used for primer and probe design in the RT-RPA-LFS assay. [file Data_Sheet_1.docx]

>KJ541383.1

AATGACCCCATTGCAAATGCAGTGGAAAGTGCTGTTAGCGCGCTTGCTGACACCACAATATCCCGGGTGACCGCAGCCAACACTGCAGTTAGTACCCACTCCCTGGGCACAGGGCGTGTACCAGCATTGCAAGCTGCGGAAACGGGAGCAAGTTCTAATGCTAGTGATGAGAACCTTATTGAGACTCGCTGTGTGATGAATCGAAACGGGGTTAATGAGGCGAGTGTGGAGCACTTTTACTCTCGTGCAGGGCTGGTAGGAGTTGTGGAGGTGAAGGACTCGGGCACTAGCCTGGATGGGTACACAGTTTGGCCCATAGATGTGATGGGCTTCGTGCAGCAGCGGCGCAAACTAGAGCTGTCAACATACATGCGCTTTGATGCCGAGTTCACTTTTGTGTCCAACCTCAGCGATAGCACAACGCCCGGGATGCTGCTGCAGTATATGTATGTACCACCAGGGGCCCCTAAGCCGGATAGCAGGAAATCATACCAATGGCAGACTGCTACTAACCCGTCGGTATTCGCAAAATTGAGTGATCCACCCCCCCAGGTGTCTGTTCCGTTCATGTCGCCAGCAACAGCTTATCAGTGGTTTTATGATGGCTACCCCACATTTGGTGAGCACAAACAAGCTACCAATTTGCAATATGGGCAGTGCCCTAACAACATGATGGGCCATTTTGCCATCCGAACTGTCAGTGAATCTACCACCGGGAAAAACATCCACGTTCGGGTGTACATGAGAATTAAGCACGTGAGAGCTTGGGTACCTAGACCCCTTCGATCCCAAGCTTATATGGTCAAGAATTACCCGACATACAGCCAAACAATAACTAACACTGCAACCGACCGTGCAAGCATAACCACCACGGATTATGAAGGCGGGGTACCAGCAAACCCACAAAGAACATCT

>KJ848301.1

AATGACCCCATTGCAAATGCAGTGGAAAGTGCTGTTAGCGCGCTTGCTGACACCACAATATCCCGGGTGACCGCAGCCAACACTGCAGTTAGTACCCACTCCCTGGGCACAGGGCGTGTACCAGCATTGCAAGCTGCGGAAACGGGAGCGAGTTCTAATGCTAGTGATGAGAACCTTATTGAGACTCGCTGTGTGATGAATCGAAACGGGGTTAATGAGGCGAGTGTGGAACACTTTTACTCTCGTGCAGGGCTGGTAGGAGTTGTGGAGGTGAAGGACTCGGGCACTAGCCTGGATGGGTACACAGTTTGGCCCATAGATGTGATGGGCTTCGTGCAGCAGCGGCGCAAACTAGAGCTGTCAACATACATGCGCTTTGATGCCGAGTTCACTTTTGTGTCCAACCTCAGTGATAGCACAACGCCCGGGATGCTGCTGCAGTATATGTATGTACCACCAGGGGCCCCTAAGCCGGATAGCAGGAAATCATACCAATGGCAGACTGCTACTAACCCGTCGGTATTCGCAAAATTGAGTGATCCACCCCCCCAGGTGTCTGTTCCGTTCATGTCGCCAGCAACAGCTTATCAGTGGTTTTATGATGGTTACCCCACATTTGGTGAGCACAAACAAGCTACCAATTTGCAATATGGGCAGTGCCCTAACAACATGATGGGCCATTTTGCCATCCGAACTGTCAGTGAATCTACCACCGGGAAAAACATCCACGTTCGGGTGTACATGAGAATTAAGCACGTGAGAGCTTGGGTACCTAGACCCCTTCGATCCCAAGCTTATATGGTCAAGAATTACCCGACATACAGCCAAACAATAACTAACACTGCAACCGACCGTGCAAGCATAACCACCACGGACTATGAAGGCGGGGTACCAGCAAACCCACAAAGAACGTCT

>KP005862.1

AATGATCCCATTACAAATGCAGTGGAAAGCGCTGTGAGCGCGCTTGCTGACACTACAATATCCCGGGTGACCGCAGCCAACACTGCAGCTAGTACCCACTCCCTGGGAACAGGGCGTGTACCAGCATTGCAAGCCGCAGAAACGGGAGCAAGCTCTAATGCTAGTGATGAGAACCTTATTGAGACTCGCTGTGTGATGAATCGAAACGGGGTTAATGAGGCGAGTGTGGAACACTTTTACTCTCGTGCAGGGCTGGTAGGAGTTGTGGAGGTGAAGGACTCGGGCACTAGCCTGGATGGGTACACAGTTTGGCCCATAGATGTGATGGGCTTCGTGCAACAGCGGCGCAAGCTAGAGCTGTCAACATACATGCGCTTTGATGCCGAGTTCACTTTTGTGTCCAACCTCAATGACAGCACGACGCCCGGGATGCTGCTGCAGTATATGTATGTACCACCAGGGGCCCCTAAGCCGGATAGCAGGAAATCATATCAATGGCAGACTGCTACTAACCCGTCGGTATTCGCAAAATTGAGTGATCCACCCCCCCAGGTATCTGTCCCGTTCATGTCGCCAGCAACAGCTTATCAGTGGTTTTATGATGGTTACCCTACATTTGGTGAGCACAAACAAGCTACCAATTTGCAATATGGGCAGTGTCCTAATAACATGATGGGTCATTTTGCCATCCGAACAGTCAGTGAATCTACCACCGGGAAAAACGTCCACGTTCGGGTGTACATGAGAATTAAGCACGTGAGAGCTTGGGTACCTAGACCCCTTCGATCCCAAGCTTATATGGTCAAGAACTACCCGACATACAGCCAAACAATAACTAACACTGCAACTGACCGTGCAAGCATAACCACCACGGATTATGAAGGCGGGGTACCAGCAAACCCACAAAGGACATCT

>KP005894.1

AATGACCCCATTGCAAATGCAGTGGAAAGTGCTGTTAGCGCGCTTGCTGACACCACAATATCCCGGGTGACCGCAGCCAACACTGCAGTTAGTACCCACTCCCTGGGCACAGGGCGTGTGCCAGCATTGCAAGCTGCGGAAACGGGAGCAAGTTCTAATGCTAGTGATGAGAACCTTATTGAGACTCGCTGTGTAATGAATCGAAACGGGGTTAATGAGGCGAGTGTGGAACACTTTTACTCTCGTGCAGGGCTGGTAGGGGTTGTGGAGGTGAAGGACTCGGGCACTAGTCTGGATGGGTACACAGTTTGGCCCATAGATGTGATGGGCTTCGTGCAGCAGCGGCGCAAACTAGAGCTGTCAACATACATGCGCTTTGATGCCGAGTTCACTTTTGTGTCCAACCTCAGTGATAGCACAACACCCGGGATGTTGCTGCAGTATATGTATGTACCACCAGGGGCCCCTAAGCCGGATAGCAGGAAATCATACCAATGGCAGACTGCTACTAACCCGTCGGTATTCGCAAAGTTGAGTGATCCACCCCCCCAGGTGTCTGTTCCGTTCATGTCGCCAGCAACAGCTTATCAGTGGTTTTATGATGGTTACCCCACATTTGGTGAGCACAAACAAGCTACCAATTTGCAATATGGGCAGTGCCCTAACAACATGATGGGCCATTTTGCCATCCGAACTGTCAGTGAATCTACCACCGGGAAAAACATCCACGTTCGGGTGTACATGAGAATTAAGCACGTGAGAGCTTGGGTACCTAGACCCCTTCGATCCCAAGCTTATATGGTCAAGAATTACCCGACATACAGCCAAACAATAACTAACACTGCAACCGACCGTGCAAGCATAACCACCACGGATTATGAAGGCGGGGTGCCAGCAAACCCACAAAGAACATCT

>KP289366.1

AATGACCCCATTGCAAATGCAGTGGAAAGTGCTGTGAGCGCGCTTGCTGACACCACAATATCCCGGGTGACCGCAGCCAATACTGCAGTTAGTACCCACTCCCTGGGCACAGGGCGTGTACCAGCATTGCAAGCTGCAGAAACGGGAGCAAGTTCTAATGCTAGTGATGAGAACCTTATTGAGACTCGCTGTGTGATGAATCGAAACGGGATTAATGAGGCGAGTGTGGAACACTTTTACTCTCGTGCAGGGCTGGTAGGAGTTGTGGAGGTGAAGGACTCGGGCACTAGCCTGGATGGGTACACAGTTTGGCCCATAGATGTGATGGGCTTCGTGCAGCAGCGGCGCAAACTAGAGCTGTCAACATACATGCGCTTTGACGCCGAGTTCACTTTTGTGTCCAACCTCAGTGATAGCACAACGCCCGGGATGCTGCTGCAGTATATGTATGTACCACCAGGGGCCCCTAAGCCGGATAGCAGGAAATCATACCAATGGCAGACTGCTACTAACCCGTCGATATTTGCAAAATTGAGTGATCCACCCCCCCAGGTGTCTGTTCCGTTCATGTCGCCAGCAACAGCTTATCAGTGGTTTTATGATGGTTACCCCACATTCGGTGAGCACAAACAAGCTACCAATTTGCAATATGGGCAGTGCCCTAACAACATGATGGGCCATTTTGCCATCCGAACTGTCAGTGAATCTACCACCGGGAAAAACATCCACGTTCGGGTGTACATGAGAATTAAGCACGTGAGAGCTTGGGTACCTAGACCCCTTCGATCCCAAGCTTATATGGTCAAGAATTACCCGACATACAGCCAAACAATAACTAACACTGCAACCGACCGTGCAAGCATAACCACCACGGATTATGAAGGCGGGGTACCAGCAAACCCACAAAGAACATCT

>KT985012.1

AACGATCCTATCACGAATGCAGTGGAAAGTGCTGTAAGCACACTCGCTGACACCACAATATCCCGGGTGACCGCAGCTAACACCGCAGCCAGTACCCACTCCCTGGGTACAGGACGTGTACCTGCACTGCAAGCTGCAGAAACGGGAGCAAGTTCTAACTCTAGTGATGAGAATCTCATCGAAACTCGCTGTGTGATGAATCGAAACGGGGTCAATGAGGCGAGCGTGGAACACTTTTACTCTCGTGCGGGGCTGGTAGGAGTTGTGGAGGTGAAGGACTCGGGCACTAGCCTAGATGGGTATACAGTGTGGCCTATAGACGTGATGGGTTTTGTGCAACAGCGGCGCAAACTAGAGCTATCAACATACATGCGCTTTGATGCTGAGTTCACCTTCGTGTCCAACCTCAATGATAGTACAACCCCTGGAATGCTATTGCAGTATATGTATGTACCACCGGGAGCCCCCAAGCCGGATAGTAGAAAATCATATCAATGGCAGACTGCTACCAACCCGTCGGTATTCGCAAAATTGAGCGATCCACCCCCCCAGGTGTCAGTCCCGTTCATGTCTCCAGCGACAGCTTATCAGTGGTTTTATGATGGTTACCCCACGTTCGGTGAGCACAAGCAAGCCACCAACTTGCAGTATGGACAGTGCCCTAATAACATGATGGGACATTTTGCCATCCGAACAGTCAGTGAGTCCACCACCGGGAAAAACATCCACGTCCGGGTGTACATGAGAATCAAACACGTGAGAGCTTGGGTGCCTAGGCCCCTCCGATCCCAAGCTTACATGGTCAAGAATTATCCAACATATAGCCAGACAATAACTAATGCTGCAACCGACCGTGCAAGCATAACCACCACGGATTATGAAGGCGGGGTACCTGCAAACCCGCAAAGAACATCT

>KU212263.1

AATGACCCCATTACAAATGCAGTGGAAAGTGCTGTTAGCGCGCTTGCTGACACCACAATATCCCGGGTGACCGCAGCCAACACTGCAGCTAGTACCCACTCCCTGGGCACAGGGCGTGTACCAGCATTGCAAGCTGCGGAAACGGGAGCAAGTTCTAATGCTAGTGATGAGAACCTTATTGAGACTCGCTGTGTGATGAATCGTAACGGGGTTAATGAGGCGAGTGTGGAGCACTTTTACTCTCGTGCAGGGCTGGTAGGAGTTGTGGAGGTGAAGGACTCGGGCACTAGCCTGGATGGGTACACAGTTTGGCCCATAGATGTGATGGGCTTCGTGCAGCAGCGGCGCAAATTAGAGCTGTCAACATACATGCGCTTTGATGCCGAGTTCACTTTTGTGTCCAACCTCAGTGATAGCACAACGCCCGGGATGCTGCTGCAGTATATGTATGTACCACCAGGGGCCCCTAAGCCAGATAGCAGGAAATCATACCAATGGCAGACTGCTACTAACCCGTCGGTGTTCGCAAAATTGAGTGATCCACCCCCCCAGGTGTCTGTTCCGTTCATGTCGCCAGCAACAGCTTATCAGTGGTTTTATGATGGCTACCCCACATTTGGTGAGCACAAACAAGCTACCAATTTGCAATATGGGCAGTGCCCTAACAACATGATGGGCCATTTTGCCATCCGAACCGTCAGTGAATCTACCACCGGGAAAAACATCCACGTTCGGGTGTACATGAGAATTAAGCACGTGAGAGCTTGGGTACCTAGACCCCTTCGATCCCAAGCTTATATGGTCAAGAATTACCCGACATACAGCCAAACAATAACTAATACTGCAACCGACCGTGCAAGCATAACCACCACGGATTATGAAGGCGGGGTACCAGCAAACTCACAGAGAACATCT

>KU708570.1

AATGATCCCATTACAAATGCAGTGGAAAGCGCTGTGAGCGCGCTTGCTGATACCACAATATCCCGGGTGACCGCAGCCAGCACTGCAGCTAGCACCCACTCCCTGGGAACAGGGCGTGTACCAGCATTGCAAGCCGCAGAAACGGGAGCAAGCTCTAATGCTAGTGATGAGAACCTTATTGAGACTCGCTGTGTGATGAATCGAAACGGGGTTAATGAAGCGAGTGTGGAACACTTTTACTCTCGAGCAGGGCTGGTAGGAGTTGTGGAGGTGAAGGACTCGGGCACTAGCCTGGATGGGTACACAGTTTGGCCCATAGATGTGATGGGCTTCGTGCAACAGCGGCGCAAACTAGAGCTGTCAACATACATGCGCTTTGATGCCGAGTTCACTTTTGTGTCCAACCTCAATGACAGCACGACGCCCGGGATGTTGCTGCAGTATATGTATGTACCACCAGGGGCCCCTAAGCCGGATAGTAGGAAATCATATCAATGGCAGACTGCTACTAACCCGTCGGTATTTGCAAAATTGAGTGATCCACCCCCCCAGGTATCTGTTCCGTTCATGTCGCCAGCAACAGCATATCAGTGGTTTTATGATGGTTACCCTACATTTGGTGAGCACAAACAAGCTACCAATTTGCAATATGGGCAGTGTCCTAATAACATGATGGGCCATTTTGCCATCCGAACAGTCAGTGAATCTACCACCGGGAAAAACGTCCACGTTCGGGTGTACATGAGAATTAAGCACGTGAGAGCTTGGGTACCTAGACCCCTTCGATCCCAAGCTTATATGGTCAAGAACTACCCGACATACAGCCAAACAATAACTAACACTGCAACTGACCGTGCAAGCATAACCACCACGGATTATGAAGGTGGGGTACCAGCAAACCCACAAAGGACATCT

>KU708573.1

AATGACCCCATTGCAAATGCAGTGGAAAGTGCTGTTAGCGCGCTTGCTGACACCACAATATCCCGGGTGACCGCAGCCAACACTGCAGTTAGTACCCACTCCCTGGGCACAGGGCGTGTACCAGCATTGCAAGCTGCGGAAACGGGAGCAAGTTCTAATGCTAGTGATGAGAACCTTATTGAGACTCGCTGTGTGATGAATCGAAACGGGGTTAATGAGGCGAGTGTGGAACACTTTTACTCTCGTGCAGGGCTGGTAGGAGTTGTGGAGGTGAAGGACTCGGGCACTAGCCCGGATGGGTACACAGTTTGGCCCATAGATGTGATGGGCTTCGTGCAGCAGCGGCGCAAACTAGAGCTGTCAACATACATGCGCTTTGATGCCGAGTTCACTTTTGTGTCCAACCTCAGTGATAGCACAACGCCCGGGATGCTGCTGCAGTATATGTATGTACCACCAGGGGCCCCTAAGCCGGATAGCAGGAAATCATACCAATGGCAGACTGCTACTAACCCGTCGGTATTCGCAAAATTGAGTGATCCACCCCCCCAGGTGTCTGTTCCGTTCATGTCGCCAGCAACAGCTTATCAGTGGTTTTATGATGGTTACCCCACATTTGGTGAGCACAAACAAGCTACCAATTTGCAATATGGGCAGTGCCCTAACAACATGATGGGCCATTTTGCCATCCGAACTGTCAGTGAATCTACCACCGGGAAAAATATCCACGTTCGGGTGTACATGAGAATTAAGCACGTGAGAGCTTGGGTACCTAGACCCCTTCGATCCCAAGCTTATATGGTCAAGAATTACCCGACATACAGCCAAACAATAACTAACACTGCAACCGACCGTGCAAGCATAACCACCACGGATTATGAAGGCGGGGTACCAGCAAACCCACAAAGAACATCT

>KU708579.1

AATGATCCCATTACAAATGCAGTGGAAAGCGCTGTGAGCGCGCTTGCTGACACCACAATATCCCGGGTGACCGCAGCCAACACTGCAGCTAGCACCCACTCCCTGGGAACAGGGCGTGTACCAGCATTGCAAGCCGCAGAAACGGGAGCAAGCTCTAATGCTAGTGATGAGAACCTTATTGAGACTCGCTGTGTGATGAATCGAAACGGGGTTAATGAGGCGAGTGTGGAACACTTTTACTCTCGTGCAGGGCTGGTAGGAGTTGTGGAGGTGAAGGACTCGGGCACTAGCCTGGATGGGTACACAGTTTGGCCCATAGATGTGATGGGCTTCGTGCAACAGCGGCGCAAGCTAGAGCTGTCAACATACATGCGCTTTGATGCCGAGTTCACTTTTGTGTCCAACCTCAATGACAGCACGACGCCCGGGATGCTGCTGCAGTATATGTATGTACCACCAGGGGCCCCTAAGCCGGATAGCAGGAAATCATATCAATGGCAGACTGCTACTAACCCGTCGGTATTCGCAAAATTGAGTGATCCACCCCCCCAGGTATCTGTCCCGTTCATGTCGCCAGCAACAGCTTATCAGTGGTTTTATGATGGTTACCCTACATTTGGTGAGCACAAACAAGCTACCAATTTGCAATATGGGCAGTGTCCTAATAACATGATGGGCCACTTTGCCATCCGAACAGTCAGTGAATCTACCACCGGGAAAAACGTCCACGTTAGGGTGTACATGAGAATTAAGCACGTGAGAGCTTGGGTACCTAGACCCCTTCGATCCCAAGCTTATATGGTCAAGAACTACCCGACATACAGCCAAACAATAACTAACACTGCAACTGACCGTGCAAGCATAACCACCACGGATTATGAAGGCGGGGTACCAGCAAACCCACAAAGGACATCT

>KU708586.1

AATGATCCCATTACAAATGCAGTGGAAAGCGCTGTGAGCGCGCTTGCTGACACCACAATATCCCGGGTGACCGCAGCCAACACTGCAGCTAGCACCCACTCCCTGGGAACAGGGCGTGTACCAGCATTGCAAGCCGCAGAAACGGGAGCAAGCTCTAATGCTAGTGATGAGAACCTTATTGAGACTCGCTGCGTGATGAATCGAAACGGGGTTAATGAGGCGAGTGTGGAACACTTTTACTCTCGTGCAGGACTGGTAGGAGTTGTGGAGGTGAAGGACTCGGGCACTAGCCTGGATGGGTACACAGTTTGGCCCATAGACGTGATGGGCTTCGTGCAACAGCGGCGCAAGCTAGAGCTGTCAACATACATGCGCTTTGATGCCGAGTTCACTTTTGTGTCCAACCTCAATGACAGCACGACGCCCGGGATGCTACTGCAGTATATGTATGTACCACCAGGGGCCCCTAAGCCGGATAGCAGGAAATCATACCAATGGCAGACTGCTACTAACCCGTCGATATTCGCAAAATTGAGTGACCCACCCCCTCAGGTATCTGTCCCGTTCATGTCGCCAGCAACAGCTTATCAGTGGTTCTATGATGGTTACCCTACATTTGGTGAGCACAAACAAGCTACCAATTTGCAATATGGGCAGTGTCCTAATAACATGATGGGCCATTTTGCCATCCGAACAGTCAGTGAATCTACCACCGGGAAAAACGTCCACGTTCGGGTGTACATGAGAATTAAGCACGTGAGAGCTTGGGTACCTAGACCCCTTCGATCCCAAGCTTATATGGTCAAAAACTACCCGACATACAGCCAAACAATAACTAACACTGCAACTGACCGTGCAAGCATAACCACCACAGATTATGAAGGCGGGGTACCAGCAAACCCACAAAGGACATCT

>KU708597.1

AATGATCCCATTACAAATGCGGTGGAAAGCGCTGTGAGCGCGCTTGCTGATACCACAATATCCCGGGTGACCGCAGCCAACACTGCAGCTAGCACCCACTCCTTGGGAACAGGGCGCGTACCAGCATTGCAAGCCGCAGAAACGGGAGCAAGTTCTAATGCTAGTGATGAGAACCTTATTGAGACTCGCTGTGTGATGAATCGAAACGGGGTTAATGAGGCGAGTGTGGAACACTTTTACTCTCGTGCAGGACTGGTAGGAGTTGTGGAGGTGAAGGATTCGGGCACTAGCCTGGATGGGTACACAGTTTGGCCCATAGACGTGATGGGCTTCGTGCAACAGCGGCGCAAGCTAGAGCTGTCAACGTACATGCGCTTTGATGCCGAGTTCACTTTTGTGTCCAACCTCAATGACAGCACGACGCCCGGGATGCTACTGCAGTATATGTATGTACCACCAGGGGCCCCTAAGCCGGATAGCAGGAAATCATACCAATGGCAGACTGCTACTAACCCGTCGATATTCGCAAAATTGAGTGACCCACCCCCTCAGGTATCTGTCCCGTTCATGTCGCCAGCAACAGCTTATCAGTGGTTCTATGATGGTTACCCTACATTTGGTGAGCACAAACAAGCTACCAACTTGCAATATGGGCAGTGTCCTAATAACATGATGGGCCATTTTGCCATCCGAACAGTTAGTGAATCTACCACCGGGAAAAACGTCCACGTTCGGGTGTACATGAGAATTAAGCACGTGAGAGCTTGGGTACCTAGACCCCTTCGATCCCAAGCTTATATGGTCAAAAACTACCCGACATACAGCCAGACAATAACTAACACTGCAACTGACCGTGCAAGCATAACCACCACAGATTATGAAGGCGGGGTGCCAGCAAACCCACAAAGGACATCT

>KX064306.1

AATGACCCCATTGCAAATGCAGTGGAAAGTGCTGTTAGCGCACTTGCTGACACCACAATATCCCGAGTGACCGCAGCCAACACTGCAGTTAGTACCCACTCCCTGGGCACAGGGCGTGTACCAGCATTGCAAGCTGCGGAAACGGGAGCAAGTTCTAATGCTAGTGATGAGAACCTTATTGAGACTCGCTGTGTGATGAATCGAAATGGGGTTAATGAGGCGAGTGTGGAGCACTTTTACTCTCGTGCAGGGCTGGTAGGAGTTGTGGAGGTAAAGGATTCGGGCACTAGCCTGGATGGGTACACAGTTTGGCCCATAGATGTGATGGGCTTCGTGCAGCAGCGGCGCAAACTAGAGCTGTCAACATATATGCGCTTTGATGCCGAGTTCACTTTTGTGTCCAACCTCAGTGATAGCACAACGCCCGGGATGCTGCTGCAGTACATGTACGTACCACCAGGGGCCCCTAAGCCGGATAGCAGGAAATCATACCAATGGCAGACTGCTACTAACCCGTCGGTATTCGCAAAATTGAGTGATCCACCCCCCCAGGTGTCTGTTCCGTTCATGTCACCAGCAACAGCTTATCAGTGGTTTTATGATGGCTACCCCACATTTGGTGAGCACAAACACGCTACCAATTTGCAATATGGGCAGTGCCCTAACAACATGATGGGCCATTTTGCCATCCGAACCGTCAGTAAATCTACCACCGGGAAAAACATCCACGTTCGGGTGTACATGAGAATTAAGCACGTGAGAGCTTGGGTACCTAGACCCCTTCGATCACAAGCTTATATGGTCAAGAATTACCCGACGTACAGCCAAACAATAACTAACACTGCAACCGACCGTGCAAGCATAACCACCACGGATTATGAAGGCGGGGTACCAGCAAACCCACAAAGAACATCT

>KX064307.1

AATGATCCCATTACAAATGCAGTGGAAAGCGCTGTGAGTGCGCTTGCTGACACCACAATATCCCGGGTGACCGCAGCCAACACTGCAGCTAGCACCCACTCCCTGGGAACAGGGCGTGTACCAGCATTGCAAGCCGCAGAAACGGGAGCAAGCTCTAATGCTAGTGATGAGAACCTTATTGAGACTCGCTGTGTGATGAATCGAAACGGGGTTAATGAGGCGAGTGTGGAACACTTTTACTCTCGTGCAGGGCTGGTAGGAGTTGTGGAGGTGAAGGACTCGGGCACTAGCCTGGATGGGTACACAGTTTGGCCCATAGATGTGATGGGCTTCGTGCAGCAGCGGCGCAAGCTAGAGCTGTCAACATACATGCGCTTTGATGCCGAGTTCACTTTTGTGTCTAACCTTGATGACAGCACGACTCCCGGGATGCTGCTACAGTATATGTATGTACCACCAGGGGCCCCTAAGCCGGATAGTAGGAAATCATATCAATGGCAGACTGCTACTAACCCGTCGGTATTCGCAAAGTTGAGTGATCCACCCCCCCAGGTATCTGTCCCGTTCATGTCGCCAGCAACAGCTTATCAGTGGTTTTATGATGGTTACCCTACATTTGGTGAGCACAAACAAGCCACCAATTTGCAATATGGGCAGTGTCCTAATAACATGATGGGCCATTTTGCCATCCGTACAGTCAGTGAATCTACCACCGGGAAAAACGTCCACGTTCGGGTGTTCATGAGAATTAAGCACGTGAGAGCTTGGGTACCTAGACCCCTTCGATCCCAAGCTTATATGGTCAAGAATTACCCGACATACAGCCAAACAATAACTAACACTGCAACTGACCGTGCAAGCATAACCACCACGGATTATGAAGGCGGGGTGCCAGCAAACCCACAAAGGACATCT

>KX189191.1

AATGACCCCATTGCAAATGCAGTGGAAGGTGCTGTGAGCGCGCTTGCTGACACCACAATATCCCGGGTGACCGCAGCCAACACTGCAGTTAGTACCCACTCCCTGGGCACAGGACGTGTACCAGCATTGCAAGCTGCAGAAACGGGAGCAAGTTCTAATGCTAGTGATGAGAACCTTATTGAGACTCGCTGTGTGATGAATCGAAACGGGGTTAATGAGGCGAGTGTGGAACACTTTTACTCTCGTGCAGGGCTGGTAGGAGTTGTGGAGGTAAAGGACTCGGGCACTAGCCTGGATGGGTACACAGTTTGGCCCATAGATGTGATGGGCTTCGTGCAGCAGCGGCGCAAACTAGAGCTGTCAACATACATGCGCTTTGATGCCGAGTTCACTTTCGTGTCCAACCTCAGTGATAGCACAACGCCCGGGATGCTGCTGCAGTATATGTATGTACCACCAGGGGCCCCTAAGCCGGATAGCAGGAAATCATACCAATGGCAGACTGCTACTAACCCGTCGGTATTTGCAAAATTGAGTGATCCACCCCCCCAGGTGTCTGTTCCGTTCATGTCGCCAGCAACAGCCTATCAGTGGTTTTATGATGGTTACCCCACATTTGGTGAGCACAAACAAGCTACCAATTTGCAATATGGGCAGTGCCCTAACAACATGATGGGCCATTTTGCCATCCGAACTGTCAGTGAATCTACCACCGGAAAAAACATCCACGTTCGGGTGTACATGAGAATTAAGCACGTGAGAGCTTGGGTGCCTAGACCCCTTCGATCCCAAGCTTATATGGTCAAGAATTATCCGACATACAGCCAAACGATAACTAACACTGCAACCGACCGTGCAAGCATAACCACCACGGATTATGAAGGCGGGGTACCAGCAAACCCACAAAGAACATCT

>KX364096.1

AATGACCCCATTACAAATGCAGTGGAAAGCGCTGTGAGCGCGCTTGCTGACACCACGATATCCCGGGTGACCGCAGCCAACACTACAGCTAGCACCCACTCCCTGGGAACAGGGCGTGTACCAGCATTGCAAGCTGCAGAAACAGGAGCAAGCTCTAATGCTAGTGATGAGAACCTTATTGAGACCCGCTGTGTGATGAATCGAAACGGGGTTAATGAGGCGAGTGTGGAACACTTTTACTCTCGTGCAGGGCTGGTAGGAGTTGTGGAGGTAAAGGACTCGGGCACTAGCCTGGATGGATACACAGTTTGGCCCATAGATGTGATGGGCTTCGTACAGCAGCGGCGCAAGCTAGAGCTGTCAACATACATGCGCTTTGATGCCGAGTTCACTTTTGTGTCCAACCTCAATGACAGCACGACGCCCGGGATGTTGCTGCAGTATATGTATGTACCACCAGGGGCCCCTAAGCCGGATAGCAGGAAATCATATCAATGGCAGACTGCTACTAACCCGTCAGTATTCGCAAAATTGAGTGATCCACCCCCCCAGGTGTCTGTTCCGTTCATGTCGCCAGCAACAGCTTATCAGTGGTTTTATGATGGTTACCCTACATTTGGCGAGCACAAACAAGCTACCAATTTGCAATATGGGCAGTGTCCTAATAACATGATGGGCCATTTTGCCATCCGAACAGTCAGTGAATCTACCACCGGGAAAAACGTTCACGTTCGGGTGTACATGAGAATTAAGCACGTGAGAGCTTGGGTACCTAGACCCCTTCGATCCCAAGCTTATATGGTCAAGAACTACCCGACATACAGCCAAACAATAACTAACACTGCAACTGACCGTGCAAGCATAACCACCACGGATTATGAAGGCGGGGTACCAGCAAACCCACAAAGGACATCT

>KX430799.1

AATGATCCCATTACAAATGCAGTGGAAAGCGCTGTGAGCGCACTTGCTGACACCACAATATCCCGGGTGACCGCAGCCAACACTGCAGCCAGTACCCACTCCCTGGGAACAGGGCGTGTACCAGCATTGCAAGCCGCAGAAACGGGAGCAAGCTCTAATGCTAGTGATGAGAACCTCATTGAGACTCGCTGCGTGATGAATCGAAACGGGGTTAATGAGGCGAGTGTGGAACACTTTTACTCTCGTGCAGGACTGGTGGGAGTTGTGGAGGTGAAGGACTCGGGCACCAGCCTGGATGGGTATACAGTTTGGCCCATAGATGTGATGGGCTTCGTGCAACAGCGGCGCAAGCTAGAACTGTCAACATACATGCGCTTTGATGCCGAGTTCACTTTTGTGTCCAACCTCAATGACAGCACGACGCCCGGGATGTTGCTGCAGTATATGTATGTACCACCAGGGGCCCCTAAGCCGGATAGCAGGAAATCATATCAATGGCAGACTGCTACTAACCCGTCGGTATTTGCAAAATTGAGTGATCCACCCCCCCAGGTATCTGTCCCGTTTATGTCGCCAGCAACAGCTTATCAGTGGTTTTATGATGGTTACCCTACATTTGGTGAGCACAAACAAGCTACCAATTTGCAATATGGGCAGTGCCCTAACAATATGATGGGCCATTTTGCCATCCGAACAGTCAGTGAATCTACCACCGGGAAAAACGTCCACGTTCGGGTGTACATGAGAATTAAGCACGTGAGAGCTTGGGTACCTAGACCCCTTCGATCTCAAGCTTATATGGTCAAGAACTACCCGACATATAGCCAAACAATAACTAACACTGCAGCTGACCGTGCAAGCATAACCACCACGGATTATGAAGGCGGGGTACCAGCGAACCCACAAAGGACATCT

>KX595286.1

AATGACCCCATCACAAATGCTGTGGAAAGTGCTGTGAGCGCGCTTGCTGACACCACAATATCCCGGGTGACCGCAGCCAGCACTGCAGTCAGCACCCACTCCCTGGGTACGGGGCGCGTACCAGCATTGCAAGCTGCAGAGACGGGAGCAAGTTCTAATGCCAGTGATGAGAACCTTATTGAGACTCGCTGTGTGATGAATCGAAACGGGGTCAATGAGGCGAGTGTGGAACACTTTTACTCTCGTGCAGGGCTGGTAGGGGTTGTGGAGGTGAAGGACTCGGGCACTAACTTGGATGGGTACACAGTTTGGCCCATAGACGTGATGGGCTTTGTGCAACAGCGGCGCAAACTAGAGCTGTCAACGTACATGCGCTTTGATGCCGAGTTCACCTTTGTGTCCAATCTCAGTGATAGCACGACGCCCGGGATGCTACTGCAGTATATGTATGTACCACCAGGGGCCCCCAAGCCGGATAGCAGGAAATCGTACCAATGGCAGACTGCTACTAACCCGTCAGTATTCGCAAAATTAAGTGATCCACCCCCCCAGGTGTCTGTCCCATTCATGTCGCCAGCAACAGCTTATCAGTGGTTTTATGATGGTTACCCTACATTTGGTGAGCACAAACAAGCCACCAATTTACAGTATGGACAGTGCCCTAACAACATGATGGGCCATTTTGCCATCCGAACAGTCAGTGAATCTACCACCGGAAAAAACGTCCACGTTCGGGTGTACATGAGAATTAAGCACGTGAGAGCTTGGGTACCTAGGCCCCTTCGGTCCCAAGCGTATATGGTCAAGAATTACCCGACATATAGCCAAACAATAACTAACACTGCAACCGACCGTGCAAGCATAACCACCACGGATTATGAGGGTGGGGTACCAGCAAACCCACAAAGAACATCT

>KX752785.1

AATGATCCCATTACAAATGCAGTGGAAAGTGCTGTGAGCGCGCTTGCTGACACCACAATATCCCGGGTGACCGCAGCCAACACTGCAGCTAGCACCCACTCCCTGGGAACAGGGCGTGTACCAGCATTGCAAGCCGCAGAAACGGGAGCAAGCTCTAATGCTAGTGATGAGAACCTTATTGAGACTCGCTGTGTGATGAATCGAAACGGGGTTAATGAGGCGAGTGTGGAACACTTTTACTCTCGTGCAGGGCTGGTAGGAGTTGTGGAGGTGAAGGACTCGGGCACTAGCCTGGATGGGTACACAGTTTGGCCCATAGATGTGATGGGCTTCGTGCAACAGCGGCGCAAGCTAGAGCTGTCAACATACATGCGCTTTGATGCCGAGTTCACTTTTGTGTCCAACCTCAATAACAGCACGACGCCCGGGATGCTGCTGCAGTATATGTATGTACCACCAGGGGCCCCTAAGCCGGATAGCAGGAAATCATATCAATGGCAGACTGCTACTAACCCGTCGGTATTCGCAAAATTGAGTGATCCACCCCCCCAGGTATCTGTTCCGTTCATGTCGCCAGCAACAGCTTATCAGTGGTTTTATGATGGTTACCCTACATTTGGTGAGCACAAACAAGCTACCAATTTGCAATATGGGCAGTGTCCTAATAATATGATGGGCCATTTTGCCATCCGAACAGTCAGTGAATCTACCACCGGGAAAAACGTCCACGTTCGGGTGTACATGAGAATTAAGCACGTGAGAGCTTGGGTACCTAGACCCCTTCGATCCCAAGCTTATATGGTCAAGAACTACCCGACATACAACCAAACAATAACTAACACTGCAACCGACCGTGCAAGCATAACCACCACGGATTATGAAGGCGGGGTACCAGCAAACCCACAAAGGACATCT

>KX871240.1

AATGATCCCATTACAAATGCAGTGGAAAGCGCTGTGAGCGCGCTTGCTGACACCACAATATCTCGGGTGACCGCAGCCAACACTGCAGCTAGCACCCACTCCCTGGGAACAGGGCGTGTACCAGCATTGCAAGCCGCAGAAACGGGAGCAAGCTCTAATGCTAGTGATGAGAACCTTATTGAGACTCGCTGTGTGATGAATCGAAACGGGGTTAATGAGGCGAGTGTGGAGCACTTTTACTCTCGTGCAGGGCTGGTAGGAGTTGTGGAGGTGAAGGACTCGGGCACTAGCCTGGATGGGTACACAGTTTGGCCCATAGATGTGATGGGTTTCGTGCAACAGCGGCGCAAGCTAGAGCTGTCAACATACATGCGCTTTGATGCCGAGTTCACTTTTGTGTCCAACCTCAATGACAGCACGACGCCCGGGATGCTGCTGCAGTATATGTATGTACCACCAGGGGCCCCTAAGCCGGACAGCAGGAAATCATATCAATGGCAGACTGCTACTAACCCGTCGGTATTCGCAAAATTGAGTGATCCACCCCCCCAGGTATCTGTTCCGTTCATGTCGCCAGCAACAGCTTATCAGTGGTTTTATGATGGTTACCCTACATTTGGTGAGCACAAACAAGCTACCAATTTGCAATATGGGCAGTGTCCTAATAACATGATGGGCCATTTTGCCATCCGAACAGTCAGTGAATCTACCACCGGGAAAAACGTCCACGTTCGGGTGTACATGAGGATTAAGCACGTGAGAGCTTGGGTACCTAGACCCCTTCGATCCCAAGCTTATATGGTCAAGAATTACCCGACATACAACCAAACAATAACTAACACTGCAACTGACCGTGCAAGCATAACCACCACGGATTATGAAGGCGGGGTACCAGCAAACCCACAAAGGACGTCT

>KX871243.1

AATGATCCCATTACAAATGCAGTAGAAAGCGCTGTGAGCGCGCTTGCTGACACCACAATATCCCGGGTGACCGCAGCCAACACTGCAGCTAGCACCCACTCTCTGGGAACAGGGCGTGTACCAGCATTGCAAGCCGCAGAAACGGGAGCAAGCTCTAATGCTAGTGATGAGAACCTTATTGAGACCCGCTGTGTGATGAATCGAAACGGGGTTAATGAGGCGAGTGTGGAACACTTTTACTCTCGTGCAGGGCTGGTAGGAGTTGTGGAGGTGAAGGACTCGGGCACTAGCCTGGATGGGTACACAGTTTGGCCCATAGATGTGATGGGCTTCGTGCAACAGCGGCGCAAGCTAGAGCTGTCAACATACATGCGCTTTGATGCCGAGTTCACTTTTGTGTCCAACCTCAATGACAGCACGACGCCCGGGATGCTGCTGCAGTATATGTATGTGCCACCAGGGGCCCCTAAGCCAGATAGCAGGAAATCATATCAATGGCAGACTGCTACTAACCCGTCGGTATTCGCAAAATTGAGTGATCCACCCCCCCAGGTATCTGTCCCGTTCATGTCGCCAGCAACGGCTTATCAGTGGTTTTACGATGGTTACCCTACATTTGGTGAGCACAAACAAGCTACCAATTTGCAATATGGGCAGTGTCCTAATAACATGATGGGCCATTTTGCCATCCGGACAGTCAGTGAATCCACCACCGGGAAAAACGTCCATGTTCGGGTGTACATGAGAATTAAGCACGTGAGAGCTTGGGTACCTAGACCCCTTCGATCCCAAGCTTATATGGTCAAGAACTATCCGACATACAGCCAAACAATAACTAACACTGCAACTGACCGTGCAAGCATAACCACCACGGATTATGAAGGCGGGGTACCAGCAAACCCACAAAGGACATCT

>KY211705.1

AATGATCCCATTACAAATGCAGTGGAAAGCGCTGTGAGCGCGCTTGCTGACACCACAATATCCCGGGTGACCGCAGCCAATACTGCAGCTAGCACCCACTCCCTGGGAACAGGGCGTGTACCAGCATTGCAAGCCGCAGAAACGGGAGCAAGCTCTAATGCTAGTGATGAGAACCTTATTGAGACTCGCTGTGTGATGAATCGAAACGGGGTTAATGAGGCGAGTGTGGAACACTTTTACTCTCGTGCAGGGCTGGTAGGAGTTGTGGAGGTGAAGGACTCGGGCACTAGCCTGGATGGGTACACAGTTTGGCCCATAGATGTGATGGGCTTCGTGCAACAGCGGCGCAAGCTAGAACTGTCAACATACATGCGCTTTGATGCCGAGTTCACTTTTGTGTCCAACCTCAATGACAGCACGACGCCCGGGATGCTGTTGCAGTATATGTATGTACCACCAGGGGCCCCTAAGCCGGATAGCAGGAAATCATATCAATGGCAGACTGCTACTAACCCGTCGGTATTCGCAAAATTGAGTGATCCACCCCCCCAGGTATCTGTCCCGTTCATGTCGCCAGCAACAGCTTATCAGTGGTTTTATGATGGTTACCCTACATTCGGTGAGCACAAACAAGCTACCAATTTGCAATACGGGCAGTGTCCTAATAACATGATGGGCCATTTCGCCATCCGAACAGTCAGTGAATCTACCACCGGGAAAAACGTCCACGTTCGGGTGTACATGAGAATTAAGCACGTGAGAGCTTGGGTACCTAGACCCCTTCGATCCCAAGCTTATATGATTAAGAACTACCCGACATACAGCCAAACAATAACTAACACTGCAACTGACCGTGCAAGCATAACCACCACGGATTATGAAGGCGGGGTACCAGCAAACCCACAAAGGACATCT

>KY211713.1

AATGATCCCATTACAAATGCAGTGGACAGCGCTGTGAGCGCGCTTGTTGACACCACAATATCCCGGGTGACCGCAGCCAACACTGCAGCTAGCACCCACTCCCTGGGAACAGGGCGTGTACCAGCATTGCAAGCCGCAGAAACGGGAGCAAGCTCTAATGCTAGTGATGAGAACCTTATTGAGACTCGCTGTGTGATGAATCGAAACGGGGTTAATGAGGCGAGTGTGGAACACTTTTACTCTCGTGCAGGGCTGGTAGGAGTTGTGGAGGTGAAGGACTCGGGCACTAGCCTGGATGGGTACACAGTTTGGCCCATAGATGTGATGGGCTTCGTGCAACAGCGGCGCAAGCTAGAGCTGTCAACATACATGCGCTTTGATGCCGAGTTCACTTTTGTGTCCAACCTCAATGACAGCACGACGCCCGGGATGCTGCTGCAGTATATGTATGTACCACCAGGGGCCCCTAAGCCGGATAGCAGGAAATCATATCAATGGCAGACTGCTACTAACCCGTCGATATTCGCAAAATTGAGTGATCCACCCCCCCAGGTATCTGTCCCGTTTATGTCGCCAGCAACAGCTTATCAGTGGTTTTATGATGGTTACCCTACATTTGGTGAGCACAAACAAGCTACCAATTTGCAATATGGGCAGTGTCCTAATAACATGATGGGCCATTTTGCCATCCGAACAGTCAGTGAATCTACCACCGGGAAAAACGTCCACGTTCGGGTGTACATGAGAATTAAGCACGTGAGAGCTTGGGTACCTAGACCCCTTCGATCCCAAGCTTATATGGTCAAGAACTACCCGACGTACGGCCAAACAATAACTAACACTGCAACTGACCGTGCAAGTATAACCACCACAGATTATGAAGGCGGGGTACCAGCAAACCCACAAAGGACATCT

>KY211727.1

AACGACCCTATTACGAATGCAGTGGAAAGTGCTGTAAGCGCGCTCGCTGACACCACAATATCCCGGGTGACTGCAGCTAACACCGCAGCCAGTACCCACTCCCTGGGTACAGGACGTGTACCTGCACTGCAAGCTGCAGAAACGGGAGCGAGTTCTAACTCTAGTGATGAGAATCTCATCGAAACTCGCTGTGTGATGAATCGAAACGGGGTCAATGAGGCGAGTGTGGAACACTTTTATTCTCGTGCAGGGCTGGTAGGAGTTGTGGAGGTGAAGGACTCGGGCACTAGCCTAGATGGGTATACAGTGTGGCCTATAGACGTGATGGGTTTCGTGCAACAGCGGCGCAAACTAGAGCTATCAACATACATGCGCTTTGACGCTGAGTTCACTTTTGTGTCCAACCTCAATGATAGTACAACCCCTGGAATGCTATTGCAGTACATGTATGTACCACCAGGAGCCCCCAAGCCGGATAGTAGAAAATCATACCAATGGCAGACTGCTACCAACCCGTCGGTGTTCGCAAAATTGAGCGATCCACCCCCCCAGGTGTCAGTCCCGTTTATGTCTCCAGCGACAGCTTATCAGTGGTTTTATGATGGTTACCCCACGTTCGGTGAACACAAGCAAGCCACTAACTTGCAGTATGGACAATGTCCTAACAACATGATGGGACATTTTGCTATCCGAACAGTCAGTGAGTCCACCACCGGGAAAAACATCCACGTTCGGGTGTACATGAGAATCAAACACGTGAGAGCTTGGGTGCCTAGGCCTCTCCGATCCCAAGCTTACATGGTCAAGAATTATCCGACATATAACCAGACAATAACTAACACCGCAACCGACCGTGCAAGCATAACCACCACGGATTATGAAGGCGGGGTACCTGCAAACCCGCAAAGAACATCT

>KY211735.1

AATGATCCCATTACAAATGCAGTGGAAAGCGCTGTGAGCGCACTTGCTGACACCACAATATCCCGGGTGACCGCAGCCAACACTGCAGCTAGCACCCACTCCCTGGGAACAGGGCGTGTACCAGCATTGCAAGCCGCAGAAACGGGAGCAAGTTCTAATGCTAGTGATGAGAACCTTATTGAGACCCGCTGTGTGATGAATCGAAACGGGGTTAATGAGGCGAGTGTGGAACACTTCTACTCTCGTGCAGGGCTGGTAGGAGTTGTGGAGGTGAAGGACTCGGGCACTAGCCTGGATGGGTACACAGTTTGGCCCATAGATGTGATGGGCTTCGTGCAACAGCGGCGCAAGCTAGAACTGTCAACATACATGCGCTTTGATGCCGAGTTCACTTTTGTGTCCAACCTCAATAACAGCACGACGCCCGGGATGCTGCTGCAGTATATGTATGTACCACCAGGGGCCCCTAAGCCGGATAGCAGGAAATCATATCAATGGCAGACTGCTACTAACCCGTCGGTATTCTCAAAATTGAGTGATCCACCCCCCCAGGTATCTGTTCCGTTCATGTCGCCAGCAACAGCTTATCAGTGGTTTTATGATGGTTACCCTACATTTGGTGAGCACAAACAAGCTACCAATTTGCAATATGGGCAGTGTCCTAATAACATGATGGGCCATTTTGCCATCCGAACAGTCAGTGAATCTACCACCGGGAAAAACGTCCACGTTCGGGTGTACATGAGAATTAAGCACGTGAGAGCTTGGGTACCTAGACCCCTTCGATCCCAAGCTTATATGGTCAAGAACTACCCGACATACAACCAAACAATAACTAACACTGCAACTGACCGTGCAAGCATAACCACCACGGATTATGAGGGCGGGGTACCAGCAAACCCACAAAGGACATCT

>KY913474.1

AATGACCCCATTGCAAATGCAGTGGAAAGTGCTGTTAGCGCGCTTGCTGACACCACAATATCCCGGATGACCGCAGCCAACACTGCAGTTAGTACCCACTCCCTGGGCACAGGGCGTGTACCAGCATTGCAAGCTGCGGAAACGGGAGCAAGTTCTAATGCTAGTGATGAGAACCTTATTGAGACTCGCTGTGTGATGAATCGAAACGGGGTTAATGAGGCGAGTGTGGAGCACTTTTACTCTCGTGCAGGGCTGGTAGGAGTTGTGGAGGTGAAGGACTCGGGCACTAGCCTGGATGGGTACACAGTTTGGCCCATAGATGTGATGGGCTTCGTGCAGCAGCGGCGCAAACTAGAGCTGTCAACATACATGCGCTTTGATGCCGAGTTCACTTTTGTGTCCAACCTCAGTAATAGCACAACGCCCGGGATGCTGCTGCAGTATATGTATGTACCACCAGGGGCCCCTAAGCCGGATAGCAGGAAATCATACCAATGGCAGACTGCTACTAACCCGTCGGTATTCGCAAAATTGAGTGATCCACCCCCCCAGGTGTCTGTTCCGTTCATGTCGCCAGCAACAGCTTATCAGTGGTTTTATGATGGCTACCCCACATTTGGTGAGCACAAACAAGCTACCAATTTGCAATATGGGCAGTGCCCTAACAACATGATGGGCCATTTTGCCATCCGAACTGTCAGTGAATCTACCACCGGGAAAAACATCCACGTTCGGGTGTACATGAGAATTAAGCACGTGAGAGCTTGGGTACCTAGACCCCTTCGATCCCAAGCTTATATGGTCAAGAATTACCCGACATACAGCCAAACAATAACTAACACTGCAACCGACCGTGCAAGCATAACCACCACGGATTATGAAGGCGGGGTACCAGCAAACCCACAAAGAACATCT

>KY913483.1

AATGACCCTATTGCAAATGCAGTGGAAAGTGCTGTTAGCGCGCTTGCTGACACCACAATATCCCGGGTGACCGCAGCCAACACTGCAGTTAGCACCCACTCCCTGGGCACAGGGCGTGCACCAGCATTGCAAGCTGCGGAAACGGGAGCAAGTTCTAATGCTAGTGATGAGAACCTTATTGAGACTCGCTGTGTGATGAATCGAAACGGGGTTAATGAGGCGAGTGTGGAGCACTTTTACTCTCGTGCAGGGCTGGTAGGAGTTGTGGAGGTGAAGGACTCGGGCACTAGCCTGGATGGGTACACAGTTTGGCCCATAGATGTGATGGGCTTCGTGCAGCAGCGGCGCAAACTAGAGCTGTCAACATATATGCGCTTTGATGCCGAGTTCACTTTTGTGTCCAACCTCAGTGATAGCACAACGCCCGGGATGCTGCTGCAGTATATGTATGTACCACCAGGGGCCCCTAAGCCGGATAGCAGGAAATCATACCAATGGCAGACTGCTACTAACCCGTCGGTATTTGCAAAATTGAGTGATCCACCCCCTCAGGTGTCTGTTCCGTTCATGTCGCCAGCAACAGCTTATCAGTGGTTTTATGATGGCTACCCCACATTTGGTGAGCACAAACAAGCTACCAATCTGCAATATGGGCAGTGCCCTAACAACATGATGGGCCATTTTGCCATCCGAACTGTCAGTGAATCTACCACCGGGAAAAACATCCACGTTCGGGTGTACATGAGAATTAAACACGTGAGAGCCTGGGTACCTAGACCCCTTCGATCCCAAGCTTATATGGTCAAGAATTACCCGACATACAGCCAAACAATAACTAACACTGCAACCGACCGTGCAAGCATAACCACCACGGATTATGAAGGCGGGGTACCAGCAAACCCACAAAGAACATCT

>KY913498.1

AATGATCCCATTACAAATGCAGTGGAAAGCGCTGTGAGCGCGCTTGCTGACACCACGATATCCCGGGTGACCGCAGCCAACACTACAGCTAGCACCCACTCCCTGGGAACAGGGCGTGTACCAGCATTGCAAGCTGCAGAAACGGGAGCAAGCTCTAATGCTAGTGATGAGAACCTTATTGAGACCCGCTGTGTGATGAATCGAAACGGGGTTAATGAGGCGAGTGTGGAACACTTTTACTCTCGTGCAGGGCTGGTAGGAGTAGTGGAGGTAAAGGACTCGGGCACTAGCCTGGATGGGTACACAGTTTGGCCCATAGATGTAATGGGCTTCGTGCAGCAGCGGCGCAAGCTAGAGCTGTCAACATACATGCGCTTTGATGCCGAGTTCACTTTTGTGTCCAACCTCAATGACAGCACGACGCCCGGGATGCTGCTGCAGTATATGTATGTACCACCAGGGGCCCCTAAGCCGGATAGCAGGAAATCATATCAATGGCAGACTGCTACTAACCCGTCAGTATTCGCAAAATTGAGTGATCCACCCCCCCAGGTGTCTGTTCCGTTCATGTCGCCAGCAACAGCTTATCAGTGGTTTTATGATGGTTACCCTACATTTGGCGAGCACAAACAAGCTACCAATTTGCAATATGGGCAGTGTCCTAATAACATGATGGGCCATTTTGCCATCCGAACAGTCAGTGAATCTACCACCGGGAAAAACGTTCACGTTCGGGTGTACATGAGAATTAAGCACGTGAGAGCTTGGGTACCTAGACCCCTTCGATCCCAAGCTTATATGGTCAAGAACTACCCAACATACAGCCAAACAATAACTAACACTGCAACTGACCGTGCAAGCATAACCACCACGGATTATGAAGGCGGGGTGCCAGCAAACCCACAAAGGACATCT

>KY972302.1

AATGATCCCATTACAAATGCAGTGGAAAGCGCTGTGAGCGCGCTTGCTGACACCACAATATCCCGAGTGACCGCAGCCAACACTGCAGCTAGCACCCACTCCCTGGGAACAGGGCGTGTACCAGCATTGCAAGCGGCAGAAACGGGAGCAAGCTCTAATGCTAGTGATGAGAACCTTATAGAGACTCGCTGTGTGATGAATCGAAACGGGGTTAATGAGGCGAGTGTGGAACACTTTTACTCTCGTGCAGGGCTGGTAGGAGTTGTGGAGGTGAAGGACTCGGGCACTAGCCTGGATGGGTACACAGTTTGGCCCATAGATGTAATGGGCTTCGTGCAACAGCGGCGCAAGCTAGAGCTGTCAACATACATGCGCTTTGATGCTGAGTTCACTTTTGTGTCCAACCTCAATGACAGTACGACGCCCGGGATGCTGCTGCAGTATATGTATGTACCACCAGGGGCCCCTAAGCCGGATAGCAGGAAATCATACCAATGGCAGACTGCTACTAACCCGTCGGTATTCGCAAAATTGAGTGATCCACCCCCCCAGGTATCTGTTCCGTTCATGTCGCCAGCAACAGCTTATCAGTGGTTTTATGATGGTTACCCTACATTTGGTGAACACAAGCAAGCTACCAATTTGCAATATGGGCAGTGTCCTAATAACATGATGGGCCATTTTGCCATTCGAACAGTCAGTGAAGCCACCACCGGGAAAAACGTCCACGTTCGGGTGTTCATGAGAATTAAGCACGTGAGAGCTTGGGTACCTAGACCCCTCCGATCCCAAGCTTATATGGTCAAGAATTACCCGACATACAGCCAAACAATAACTAACACTGCAACCGACCGTGCAAGCATAACCACCACGGATTATGAAGGCGGGGTACCAGCAAACCCACAAAGGACATCT

>LC126150.1

AATGATCCCATTGCAAATGCAGTGGAAAGTGCTGTGAGCGCGCTTGCTGATACCACAATATCCCGGGTGACCGCAGCCAGTACTGCAGTTAGCACCCACTCCCTGGGCACAGGGCGTGTACCAGCATTGCAAGCTGCAGAAACGGGAGCAAGCTCTAATGCTAGTGATGAGAACCTTATTGAGACTCGCTGTGTGATGAATCGAAATGGGGTCAATGAGGCGAGTGTGGAACATTTTTACTCTCGCGCAGGGCTAGTAGGAGTTGTGGAGGTGAAGGACTCGGGCACTAGCCTGGATGGGTACACAGTTTGGCCCATAGACGTGATGGGCTTCGTGCAGCAGCGGCGCAAACTAGAGCTGTCAACATACATGCGCTTTGATGCCGAGTTTACTTTTGTGTCCAACCTCAATGATAGCACGACGCCCGGGATGTTGCTGCAGTATATGTATGTGCCACCAGGGGCCCCTAAACCGGATAGCAGAAAATCATACCAATGGCAGACTGCTACTAACCCGTCGATATTCGCAAAATTGAGTGATCCACCCCCCCAGGTGTCTGTCCCGTTCATGTCGCCAGCAACAGCTTATCAGTGGTTTTATGATGGTTACCCTACATTTGGTGAGCACAAGCAAGCCACCAATTTGCAATATGGGCAGTGTCCTAACAACATGATGGGCCATTTTGCCATCCGAACAGTTAGTGAATCTACCACCGGAAAAAACGTCCACGTTCGGGTGTACATGAGAATTAAGCACGTGAGAGCTTGGGTACCTAGACCCCTTCGATCCCAAGCTTATATGGTCAAGAACTACCCGACATACAGCCAAACAATAACTAACACTGCAACCGACCGTGCAAGCATAACCACCACGGATTATGAAGGCGGGGTACCAGCAAACCCACAAAGAACATCT

>LC224154.1

AATGATCCCATCACAAATGCTGTGGAAAGTGCTGTGAGCGCGCTTGCTGACACCACAATATCCCGGGTGACCGCAGCCAGCACTGCAGCCAGCACCCACTCTCTGGGTACGGGGCGCGTACCAGCATTGCAAGCTGCAGAGACGGGAGCAAGTTCTAATGCCAGTGATGAGAACCTTATTGAGACTCGCTGTGTGATGAATCGAAACGGGGTCAATGAGGCGAGTGTGGAGCACTTTTACTCTCGTGCAGGGCTGGTAGGGGTTGTGGAGGTGAAGGACTCGGGCACTAGCTTAGATGGGTACACAGTTTGGCCCATAGATGTGATGGGCTTTGTACAACAGCGGCGCAAACTAGAGCTGTCAACGTACATGCGCTTTGATGCCGAGTTCACTTTTGTATCCAACCTCAGTGATAGCACGACGCCCGGGATGCTGCTGCAGTATATGTATGTACCACCAGGGGCCCCCAAGCCGGATAGCAGGAAATCATACCAATGGCAGACTGCTACTAACCCGTCAGTATTCGCAAAATTAAGTGATCCACCCCCCCAGGTGTCTGTCCCGTTCATGTCGCCAGCAACAGCTTATCAGTGGTTTTACGATGGTTACCCTACATTTGGTGAGCACAAACAAGCCGCTAATTTACAGTATGGACAGTGCCCTAACAACATGATGGGCCATTTTGCCATCCGAACAGTCAGTGAGTCTACCACCGGAAAAAACGTCCATGTTCGGGTGTACATGAGGATTAAGCACGTGAGAGCCTGGGTACCTAGACCCCTTCGGTCTCAGGCGTATATGGTCAAGAATTACCCGACATATAGCCAAACAATAACTAACACTGCAACCGACCGTGCAAGCATAACCACCACGGATTATGAAGGTGGGGTACCAGCAAACCCACAAAGAACATCT

>LC364106.1

AATGATCCCATTACAAATGCTGTGGAAAGTGCTGTGAGCGCGCTTGCTGACACCACAATATCCCGGGTGACCGCAGCCAGCACTGCAGTCAGCACCCACTCCCTGGGTACGGGGCGCGTACCAGCATTGCAAGCTGCAGAGACGGGAGCAAGTTCTAATGCTAGTGATGAGAACCTTATTGAGACTCGCTGTGTGATGAATCGAAACGGAGTCAATGAGGCGAGTGTGGAACACTTTTACTCTCGTGCAGGGCTGGTAGGGGTTGTGGAGGTGAAGGACTCGGGCACTAGCTTGGATGGGTACACAGTTTGGCCCATAGACGTGATGGGTTTTGTGCAACAGCGGCGCAAACTAGAGCTGTCAACGTACATGCGCTTTGATGCCGAGTTCACTTTTGTGTCCAACCTCAGTGATAGCACAACGCCTGGGATGCTGCTGCAGTATATGTATGTACCACCAGGGGCCCCCAAACCGGATAGCAGGAAATCATACCAATGGCAGACTGCTACTAACCCGTCAGTATTCGCAAAATTAAGTGATCCACCCCCCCAAGTGTCTGTTCCATTCATGTCGCCAGCAACAGCTTATCAGTGGTTTTATGATGGTTATCCTACATTTGGTGAGCACAAACAAGCCACTAATTTACAGTATGGGCAGTGCCCTAACAACATGATGGGCCATTTTGCCATCCGAACAGTTAGTGAATCTACCACCGGAAAAAACGTCCACGTTCGGGTGTACATGAGAATTAAACACGTGAGAGCTTGGGTACCTAGACCCCTTCGGTCCCAAGCGTATATGGTCAAGAATTACCCGACATATAGCCAAACAATAACTAACACTGCAACTGACCGTGCAAGCATAACCACCACGGATTATGAAGGCGGGGTACCAGCAAACCCACAAAGAACATCT

>LC364136.1

AATGATCCTATTACAAATGCTGTGGAAAGTGCTGTGAGCGCGCTTGCTGACACCACAATATCCCGGGTGACCGCAGCCAACACTGCAGTCAGCACCCACTCTCTGGGTACGGGGCGCGTACCAGCATTGCAAGCTGCAGAGACGGGAGCAAGTTCTAATGCCAGTGATGAGAACCTTATTGAGACTCGCTGTGTGATGAATCGAAACGGGGTCAATGAGGCGAGTGTGGAACACTTTTACTCTCGTGCAGGGCTGGTAGGGGTTGTGGAGGTGAAGGACTCGGGCACTAGCTTGGATGGGTACACAGTTTGGCCCATAGATGTGATGGGCTTTGTGCAACAGCGGCGCAAACTAGAGCTGTCAACGTACATGCGCTTTGATGCCGAGTTCACTTTTGTATCCAACCTCAGTGATAGCACCACGCCCGGGATGCTGCTACAGTATATGTATGTACCACCAGGGGCCCCCAAGCCGGATAGCAGGAAATCATACCAATGGCAGACTGCTACTAACCCGTCAGTATTCGCAAAATTAAGTGATCCACCCCCCCAGGTGTCTGTCCCGTTCATGTCACCAGCAACAGCTTATCAGTGGTTTTATGATGGTTACCCTACGTTTGGTGAGCACAAACAAGCCACCAATTTACAGTATGGACAGTGCCCTAACAACATGATGGGCCATTTTGCCATCCGAACAGTCAGTGAATCTACCACCGGAAAAAACGTCCACGTTCGGGTGTACATGAGAATTAAGCACGTGAGAGCTTGGGTACCTAGACCCCTTCGGTCCCAAGCGTATATGGTCAAGAACTACCCGACATATAGCCAAACAATAGCTAACACTGCAACCGACCGTGCAAGCATAACCACCACAGATTATGAAGGCGGGGTACCAGCAAACCCACAAAGAACATCT

>LC364162.1

AATGATCCCATTACGAATGCTGTGGAAAGTGCTGTGAGCGCGCTTGCTGACACCACAATATCCCGGGTGACCGCAGCCAGCACTGCAGTCAGCACCCACTCTCTGGGTACGGGGCGCGTACCAGCATTGCAAGCTGCAGAGACGGGAGCAAGTTCTAATGCCAGTGATGAGAATCTTATTGAGACTCGCTGTGTGATGAATCGAAACGGGGTCAATGAGGCGAGTGTGGAACACTTTTACTCTCGTGCAGGGCTGGTAGGGGTTGTGGAGGTGAAGGACTCGGGCACTAGCTTGGATGGGTACACAGTTTGGCCCATAGATGTGATGGGCTTTGTACAACAGCGGCGCAAACTAGAGCTATCAACGTACATGCGCTTTGATGCCGAGTTCACTTTTGTATCTAACCTCAGTGATAGCACGACGCCCGGGATGCTGCTGCAGTATATGTATGTACCACCAGGGGCTCCCAAACCGGATAGCAGGAAATCATACCAATGGCAGACTGCTACTAACCCGTCAGTATTCGCAAAATTAAGTGACCCACCCCCCCAGGTGTCTGTCCCGTTCATGTCGCCAGCAACAGCTTATCAGTGGTTTTATGATGGTTACCCTACTTTTGGTGAACACAAACAAGCCACTAATTTACAGTATGGACAGTGCCCTAACAACATGATGGGCCATTTTGCCATCCGAACAGTCAGTGAATCTACCACCGGAAAAAACGTCCACGTTCGGGTGTACATGAGAATTAAGCATGTGAGAGCTTGGGTACCTAGACCCCTTCGGTCCCAAGCGTATATGGTCAAGAATTACCCGACATATAGCCAAACAATAACTAACACTGCAACCGACCGTGCAAGCATAACCACCACGGATTATGAAGGCGGGGTACCAGCAAACCCACAAAGAACATCT

>LC364172.1

AACGATCCCATTACAAGTGCAGTGGAAAGCGCTGTGAGCGCGCTTGCTGACACCACAATATCCCGGGTGACCGCAGCTAACACTGCAGCTAGCACCCACTCCTTGGGAACAGGGCGTGTACCAGCGCTGCAAGCTGCAGAAACGGGAGCGAGCTCTAATGCTAGTGATGAGAATCTTATTGAAACCCGCTGCGTGATGAATCGAAACGGGGTTAATGAAGCGAGTGTAGAGCACTTTTACTCTCGTGCAGGGCTGGTTGGAGTTGTGGAGGTGAAGGACTCGGGCACTAGCCTGGATGGGTACACAGTTTGGCCCATAGACGTGATGGGCTTCGTGCAACAGCGGCGTAAATTAGAGCTGTCAACATATATGCGCTTTGATGCCGAGTTCACCTTTGTGTCCAACCTCAATGACAGTACGACGCCCGGGATGCTGCTGCAGTATATGTATGTACCACCAGGGGCCCCTAAGCCGGATAGCAGGAAATCATACCAATGGCAGACTGCTACTAACCCGTCGGTATTCGCAAAATTGAGTGATCCACCCCCTCAGGTGTCTGTCCCGTTCATGTCGCCGGCAACAGCTTATCAGTGGTTTTATGACGGTTACCCTACATTTGGTGAGCACAAACAAGCTACCAATTTGCAATATGGGCAGTGCCCTAACAACATGATGGGCCATTTTGCCATCCGAACAGTTAGTGAATCTACCACCGGGAAAAACGTCCACGTTCGGGTGTACATGAGAATTAAGCACGTGAGGACTTGGGTACCTAGACCCCTTCGATCCCAAGCTTATATGGTCAAGAATTACCCGACATACAGCCAAACAATAACTAACACTGCAACCGACCGCGCAAGTATAACCACCACGGATTATGAAGGCGGGGTACCAGCAAACCCACAAAGAACATCT

>LC412950.1

AATGATCCCATCACAAGTGCAGTGGAAAGCGCTGTGAGCGCGCTTGCCGACACCACGATATCCCGGGTGACCGCAGCCAACACTACAGCTAGCACCCACTCCCTGGGAACAGGGCGTGTGCCAGCATTACAAGCTGCAGAAACGGGAGCAAGCTCCAATGCTAGTGATGAGAACCTTATTGAGACCCGCTGTGTGATGAATCGAAACGGGGTTAATGAGGCGAGTGTGGAACACTTTTACTCTCGTGCAGGGCTAGTAGGAGTTGTGGAGGTAAAGGACTCGGGCACTAGCCTGGATGGGTACACAGTTTGGCCCATAGATGTGATGGGCTTCGTGCAGCAGCGGCGCAAGCTAGAGCTGTCGACATACATGCGCTTTGATGCCGAGTTCACTTTTGTGTCCAACCTCAGTAACAGCACGACGCCTGGGATGCTGCTGCAGTATATGTATGTACCACCAGGAGCCCCTAAGCCGGACAGCAGGAAATCATATCAATGGCAGACTGCTACCAACCCGTCAGTATTCGCAAAATTGAGTGATCCACCCCCCCAGGTGTCTGTTCCGTTCATGTCGCCAGCAACAGCTTATCAGTGGTTTTATGATGGTTACCCTACATTTGGCGAGCACAAACAAGCTACCAATTTGCAATATGGGCAGTGTCCTAATAACATGATGGGCCATTTTGCTATCCGAACAGTCAGTGAATCTACCACCGGGAAAAACGTTCACGTTCGGGTGTACATGAGAATTAAGCACGTGAGAGCTTGGGTACCTAGACCCCTTCGATCCCAAGCTTATATGGTCAAGAACTACCCGACATACAGCCAAACAATAACTAACACTGCAACTGACCGTGCAAGCATAACCACCACGGATTATGAAGGCGGGGTACCAGCAAACCCACAAAGGACATCT

>LC413133.1

AATGATCCCATTACAAATGCAGTGGAAAGCGCTGTGAGCGCGCTTGCTGACACCACAATATCCCGGGTGACCGCAGCCAGTACTGCAGCTAGCACCCACTCCCTGGGAACAGGGCGTGTACCAGCATTGCAAGCCGCAGAAACGGGAGCAAGCTCTAATGCTAGTGATGAGAACCTTATTGAGACTCGCTGTGTGATGAATCGAAACGGGGTTAATGAGGCGAGTGTGGAACACTTTTACTCTCGTGCAGGGCTGGTAGGAGTTGTGGAGGTGAAGGACTCGGGCACTAGCTTGGATGGGTACACAGTTTGGCCCATAGATGTGATGGGCTTTGTGCAACAGCGGCGCAAGCTAGAGCTGTCAACATATATGCGCTTTGATGCCGAGTTCACTTTTGTGTCCAACCTCAATGACAGCACGACGCCTGGGATGCTGCTGCAGTACATGTATGTACCACCAGGGGCCCCTAAGCCGGATAGCAGGAAATCATATCAATGGCAGACTGCTACTAACCCGTCAGTATTTGCAAAATTGAGTGATCCACCCCCCCAAGTATCTGTTCCGTTCATGTCGCCAGCAACAGCTTATCAGTGGTTTTATGATGGTTACCCTACATTTGGTGAGCACAAACAAGCTACCAATTTGCAATATGGGCAGTGTCCTAACAACATGATGGGCCATTTTGCCATCCGAACAGTCAGTGAATCTACCACCGGGAAAAACGTCCACGTTCGGGTGTACATGAGAATTAAGCACGTGAGAGCTTGGGTACCTAGACCCCTTCGATCCCAAGCTTATATGGTCAAGAACTACCCGACATACAGCCAAACAATAACTAACACTGCAACTGACCGTGCAAGCATAACCACCACGGATTATGAAGGTGGGGTACCAGCAAACCCACAAAGGACATCT

>LC419998.1

AATGATCCCATTACGAATGCTGTGGAAAGTGCTGTGAGCGCACTTGCTGACACCACAATATCCCGGGTGACCGCAGCCAGCACTACAGTCAGCACCCACTCTCTGGGTACGGGGCGCGTACCAGCATTGCAAGCTGCAGAGACGGGAGCAAGTTCTAATGCCAGTGATGAGAATCTTATTGAGACTCGCTGTGTGATGAATCGAAACGGGGTCAATGAGGCGAGTGTGGAACACTTTTACTCTCGTGCAGGGCTGGTAGGGGTTGTGGAGGTGAAGGACTCGGGCACTAGCTTGGATGGGTACACAGTTTGGCCCATAGATGTGATGGGCTTTGTACAACAGCGGCGCAAACTAGAGCTGTCAACATACATGCGCTTTGATGCCGAGTTCACTTTTGTATCTAACCTCAGTGATAGCACGACGCCCGGGATGCTGCTGCAGTATATGTATGTACCACCAGGGGCTCCCAAACCGGATAGCAGGAAATCATACCAATGGCAGACTGCTACTAACCCGTCAGTATTCGCAAAATTAAGTGACCCACCCCCCCAGGTGTCTGTCCCGTTCATGTCGCCAGCAACAGCTTATCAGTGGTTTTATGATGGTTACCCTACTTTTGGTGAACACAAACAAGCCACTAATTTACAGTATGGACAGTGCCCTAACAACATGATGGGCCATTTTGCCATCCGAACAGTCAGTGAATCTACCACCGGAAAAAACGTCCACGTTCGGGTGTACATGAGAATTAAGCATGTGAGAGCTTGGGTACCTAGACCCCTTCGGTCCCAAGCGTATATGGTCAAGAATTACCCGACATATAGCCAAACAATAACTAACACTGCAACCGACCGTGCAAGCATAACCACCACGGATTATGAAGGCGGGGTACCAGCAAACCCACAAAGAACATCT

>LC481405.1

AATGATCCCATTACAAATGCAGTGGAAAGCGCTGTGAGTGCGCTTGCTGACACCACAATATCCCGGGTGACCGCAGCCAACACTGTAGCTAGCACCCACTCCCTGGGAACAGGGCGTGTACCAGCATTGCAAGCCGCAGAAACGGGAGCAAGCTCTAATGCTAGTGATGAGAACCTTATTGAGACTCGCTGTGTGATGAATCGAAACGGGGTTAATGAGGCGAGTGTGGAACATTTTTACTCTCGTGCAGGGCTGGTAGGAGTTGTGGAGGTGAAGGACTCGGGCACTAGCCTGGATGGGTATACAGTTTGGCCCATAGATGTGATGGGCTTCGTGCAACAGCGGCGCAAGCTAGAGCTGTCAACATACATGCGCTTTGATGCCGAGTTCACTTTTGTGTCCAACCTTAATGACAGCACGACACCCGGGATGCTACTGCAGTATATGTATGTGCCACCAGGGGCCCCTAAGCCAGATAGCAGGAAATCATATCAATGGCAGACTGCTACCAACCCATCGGTATTCGCAAAATTGAGTGATCCACCCCCCCAGGTATCTGTCCCGTTCATGTCGCCAGCAACAGCTTATCAGTGGTTTTATGATGGTTACCCTACATTTGGTGAGCACAAGCAAGCTACCAATTTGCAATATGGGCAGTGTCCTAATAACATGATGGGTCATTTTGCCATCCGAACAGTCAGTGAATCTACCACTGGGAAAAACGTCCACGTTCGGGTGTACATGAGAATTAAGCACGTGAGAGCTTGGGTACCTAGACCCCTTCGATCCCAAGCTTATATGGTCAAAAACTACCCGACATACAGCCAAACAATAACAAACACTGCAGCTGACCGTGCAAGTATAACCACTACGGATTATGAAGGCGGGGTACCAGCAAACCCACAAAGGACGTCT

>MF285634.1

AATGATCCCATTACAAATGCAGTGGAAAGCGCTGTGAGCGCGCTTGCTGACACCACAATATCCCGGGTGACCGCAGCCAACACTGCAGCTAGCACCCACTCCCTGGGAACAGGGCGTGTACCAGCATTGCAAGCCGCAGAAACAGGAGCAAGCTCTAATGCTAGTGATGAGAACCTTATTGAGACTCGCTGTGTGATGAATCGAAACGGGGTTAATGAGGCGAGTGTGGAACACTTTTACTCTCGTGCAGGGCTGGTAGGAGTTGTGGAGGTGAAGGACTCGGGTACTAGCCTGGATGGATACACAGTTTGGCCCATAGATGTGATGGGCTTCGTGCAACAGCGGCGCAAGCTAGAGCTGTCAACATACATGCGCTTTGATGCCGAGTTCACTTTTGTATCCAACCTCAATGATAGCACGACGCCCGGGATGCTCCTGCAGTATATGTATGTACCACCAGGGGCCCCTAAGCCGGATAGCAGGAAATCATATCAATGGCAGACTGCTACTAACCCGTCGGTATTCGCAAAATTGAGTGATCCACCCCCTCAGGTGTCTGTCCCGTTCATGTCGCCAGCAACAGCTTATCAGTGGTTTTATGATGGTTACCCTACATTTGGTGAGCACAAACAAGCTACCAATTTGCAATATGGGCAGTGTCCTAATAACATGATGGGCCATTTTGCCATCCGAACAGTCAGTGAATCTACCACCGGGAAAAACGTCCACGTTCGGGTGTACATGAGAATTAAGCACGTAAGAGCTTGGGTACCTAGACCCCTTCGATCCCAAGCTTATATGGTCAAGAACTACCCGACATACAGCCAAACAATAACTAACACTGCAACTGACCGTGCAAGCATAACCACCACGGATTATGAAGGCGGGGTGCCAGCAAACCCACAAAGGACGTCT

>MF285636.1

AATGATCCCATTACAAATGCAGTGGAAAGCGCTGTGAGCGCGCTTGCTGACGCCACAATATCCCGGGCGACCGCAGCCAACACTGCAGCTAGCACCCACTCCCTGGGAACAGGGCGTGTACCAGCATTGCAAGCCGCAGAAACGGGAGCAAGCTCTAATGCTAGTGATGAGAACCTTATTGAGACTCGCTGTGTGATGAATCGAAACGGGGTTAATGAGGCGAGTGTGGAACACTTTTACTCTCGTGCAGGGCTGGTAGGAGTTGTGGAGGTGAAGGACTCGGGCACTAGCCTGGATGGGTACACAGTTTGGCCCATAGATGTGATGGGCTTCGTGCAACAGCGGCGCAAGCTAGAGCTGTCAACATACATGCGCTTTGATGCCGAGTTCACTTTTGTGTCCAACCTCAATGACAGCACGACGCCCGGGATGCTGCTGCAATATATGTATGTACCACCAGGGGCCCCTAAGCCGGATAGCAGGAAATCATATCAATGGCAGACTGCTACTAACCCGTCGGTATTCGCAAAATTGAGTGATCCACCTCCCCAGGTATCTGTTCCGTTCATGTCGCCAGCAACAGCTTATCAGTGGTTTTATGATGGTTACCCTACATTTGGTGAGCACAAACAAGCTACCAATTTGCAATATGGGCAGTGTCCTAATAACATGATGGGCCATTTTGCCATCCGAACAGTCAGTGAATCTACCACCGGGAAAAACGTCCACGTTCGGGTGTACATGAGAATTAAGCACGTGAGAGCTTGGGTACCTAGACCCCTTCGATCCCAAGCTTATATGGTTAAGAACTACCCGACATACAGCCAAACAATAACTAACACTGCAACTGACCGTGCAAACATAACCACCACGGATTATGAAGGCGGGGTACCAGCAAACCCACAAAGGACATCT

>MF285658.1

AATGATCCCATTACAAATGCAGTGGAAAGCGCTGTAAGTGCGCTTGCTGACACTACAATATCCCGGGTGACCGCAGCCAACACTGCAGCTAGCACCCACTCCCTGGGAACAGGGCGTGTACCAGCATTGCAAGCCGCAGAAACGGGAGCAAGCTCTAATGCTAGTGATGAGAACCTTATTGAGACTCGCTGTGTGATGAATCGAAACGGGGTTAATGAGGCGAGTGTGGAACACTTTTACTCTCGTGCAGGGCTGGTAGGAGTTGTGGAGGTGAAGGACTCGGGCACTAGCCTGGATGGGTACACAGTTTGGCCTATAGATGTGATGGGCTTCGTGCAACAGCGGCGCAAGCTAGAGCTGTCAACATACATGCGCTTTGATGCCGAGTTCACTTTTGTGTCTAACCTTGATGACAGCACGACGCCCGGGATGCTGCTACAGTATATGTATGTACCACCAGGGGCCCCTAAGCCGGATAGCAGGAAATCGTATCAATGGCAGACTGCTACTAACCCGTCGGTATTCGCAAAGTTGAGTGATCCACCCCCCCAGGTATCTGTCCCGTTCATGTCGCCAGCAACAGCTTATCAGTGGTTTTATGATGGTTACCCTACATTTGGTGAGCACAAACAAGCCACCAATTTGCAATATGGGCAGTGTCCTAATAACATGATGGGCCATTTTGCCATCCGTACAGTCAGTGAATCTACCACCGGGAAAAACGTCCACGTTCGGGTGTTCATGAGAATTAAGCACGTGAAAGCTTGGGTACCTAGACCCCTTCGATCCCAAGCTTATATGGTCAAGAATTACCCGACATACAGCCAAACAATAACTAACACTGCAACTGACCGTGCAAGCATAACCACCACGGATTATGAAGGCGGGGTGCCAGCAAACCCACAAAGGACATCT

>MF285675.1

AATGATCCTATTACGAATGCTGTGGAAAGTGCTGTGAGCGCGCTTGCTGACACCACAATATCCCGGGTGACCGCAGCCAACACTGCAGTCAGCACCCACTCTCTGGGTACGGGGCGCGTACCAGCATTGCAAGCTGCAGAGACGGGAGCAAGCTCTAATGCTAGTGATGAGAACCTTATTGAGACTCGCTGTGTGATGAATCGAAACGGGGTCAATGAGGCGAGTGTGGAACACTTTTACTCTCGTGCAGGGCTGGTAGGGGTTGTGGAGGTGAAGGACTCGGGCACTAGCTTGGATGGGTACACAGTTTGGCCCATAGATGTGATGGGCTTTGTGCAACAGCGGCGCAAACTAGAGCTGTCAACGTACATGCGCTTTGATGCCGAGTTCACTTTTGTGTCTAACCTTAGTGATAGCACGACGCCCGGGATGCTGCTGCAGTATATGTATGTACCACCAGGGGCCCCCAAGCCGGATAGTAGGAAATCATACCAATGGCAGACTGCTACTAACCCGTCAGTATTCGCAAAATTAAGTGATCCACCCCCCCAGGTGTCTGTCCCGTTTATGTCGCCAGCAACAGCTTATCAGTGGTTTTATGATGGTTACCCTACGTTTGGTGAGCACAAACAAGCCACCAATTTACAGTATGGACAGTGCCCCAACAACATGATGGGCCATTTTGCCATCCGAACAGTCAGTGAATCTACCACCGGAAAAAACGTCCACGTACGGGTGTACATGAGAATTAAGCACGTGAGAGCTTGGGTACCTAGACCCCTTCGATCCCAAGCATATATGGTCAAGAACTACCCGACATATAGCCAAACAATAACTAACACTGCAACCGACCGTGCAAGCATAACCACCACAGATTATGAAGGCGGGGTACCAGCAAACCCACAAAGAACATCT

>MF578285.1

AATGATCCCATTACAAATGCAGTGGAAAGCGCTGTGAGCGCGCTTGCTGACACCACAATATCCCGGGTGACCGCAGCTAACACTGCAGCTAGCACCCACTCCCTGGGAACAGGGCGTGTACCAGCATTGCAAGCCGCAGAAACGGGGGCAAGCTCTAATGCTAGTGATGAGAACCTTATTGAGACTCGCTGTGTGATGAATCGAAACGGGGTTAATGAGGCGAGTGTGGAACACTTTTACTCTCGTGCAGGGCTGGTAGGAGTTGTGGAGGTGAAGGACTCGGGCACTAGCCTGGATGGGTACACAGTTTGGCCCATAGATGTGATGGGCTTCGTGCAACAGCGGCGCAAGCTAGAGCTGTCAACATACATGCGCTTTGATGCCGAGTTCACTTTTGTGTCCAACCTCAATGATAGCACGACGCCCGGGATGCTGCTGCAGTATATGTATGTACCACCAGGGGCCCCTAAGCCGGATAGCAGGAAATCATATCAATGGCAGACTGCTACTAACCCGTCGGTATTCGCAAAATTGAGTGATCCACCCCCCCAGGTATCTGTCCCGTTCATGTCGCCAGCAACAGCTTATCAGTGGTTTTATGATGGTTACCCTACATTTGGTGAGCACAAACAAGCTACCAATTTGCAATATGGGCAGTGCCCTAACAACATGATGGGCCATTTTGCCATCCGAACAGTCAGTGAATCTACCACCGGGAAAAACGTCCACGTTCGGGTGTACATGAGAATTAAGCACGTGAGAGCTTGGGTACCTAGACCCCTTCGATCCCAAGCTTATATGGTCAAGAATTACCCGACATACAGCCAAACAATAACTAACACTGCAACCGATCGTGCAAGCATAACCACCACGGATTATGAAGGCGGGGTACCAGCAAACCCACAAAGGACATCT

>MF596071.1

AATGACCCCATTGCAAATGCAGTGGAAAATGCTGTGAGCGCGCTTGCTGACACCACAATATCCCGGGTGACCGCAGCCAACACTGCAGTTAGTACCCACTCCCTGGGCACAGGGCGTGTGCCAGCATTGCAAGCTGCAGAAACGGGAGCAAGTTCTAATGCTAGTGATGAGAACCTTATTGAGACTCGCTGTGTGATGAATCGAAACGGGGTTAATGAGGCGAGTGTGGAACACTTTTACTCTCGTGCAGGGCTGGTAGGAGTTGTGGAGGTGAAGGACTCGGGCACTAGCCTGGATGGGTACACAGTTTGGCCCGTAGATGTGATGGGCTTCGTGCAGCAGCGGCGCAAACTAGAACTGTCAACATACATGCGCTTTGATGCCGAGTTCACTTTTGTGTCCAACCTCAATGATAGCACAACGCCCGGGATGCTGCTGCAGTATATGTATGTACCACCAGGGGCCCCTAAGCCGGATAGCAGGAAATCATACCAATGGCAGACTGCTACTAACCCGTCGGTATTTGCAAAATTGAGTGATCCACCCCCCCAGGTGTCTGTTCCGTTCATGTCGCCAGCAACAGCTTATCAGTGGTTTTATGATGGTTACCCCACATTTGGTGAGCACAAACAAGCTACCAATTTGCAATATGGGCAGTGCCCTAACAACATGATGGGCCATTTTGCCATCCGAACTGTTAGTGAGTCTACCACCGGGAAAAACGTCCACGTTCGGGTGTACATGAGAATTAAGCACGTGAGAGCTTGGGTACCTAGACCCCTTCGATCCCAAGCTTATATGGTGAAAAATTACCCGACATACAGCCAAACAATAACTAACACTGCAACCGACCGTGCAAGCATAACCACCACGGATTATGAAGGCGGGGTACCAGCAAATCCACAAAGAACATCT

>MF596084.1

AATGATCCCATTACAAATGCAGTGGAAAGCGCTGTGAGCGCGCTTGCTGACACCACAATATCCCGGGTGACCGCAGCCAACACTGCAGCTAGCACCCACTCCCTGGGAACAGGGCGTGTACCAGCATTGCAAGCCGCAGAAACAGGAGCAAGCTCTAATGCTAGTGATGAGAACCTTATTGAGACTCGCTGTGTGATGAATCGAAACGGGGTTAATGAGGCGAGTGTGGAACACTTTTACTCTCGTGCAGGACTGGTAGGAGTTGTGGAGGTGAAGGACTCGGGCACTAGCCTGGATGGGTACACAGTTTGGCCCATAGATGTGATGGGCTTCGTGCAACAGCGGCGCAAGCTAGAGCTGTCAACATACATGCGCTTTGATGCCGAGTTCACTTTTGTATCCAACCTCAATGATAGCACGACGCCCGGGATGCTCCTGCAGTATATGTATGTACCACCAGGGGCCCCTAAGCCGGATAGCAGGAAATCATATCAATGGCAGACTGCTACTAACCCGTCGGTATTCGCAAAATTGAGTGATCCACCCCCCCAGGTGTCTGTCCCGTTCATGTCGCCAGCAACAGCTTATCAGTGGTTTTATGATGGTTACCCTACATTTGGTGAGCACAAACAAGCTACCAATTTGCAATATGGGCAGTGTCCTAATAACATGATGGGCCATTTTGCCATCCGAACAGTCAGTGAATCTACCACCGGGAAAAACGTTCACGTTCGGGTGTACATGAGAATTAAGCACGTAAGAGCTTGGGTACCTAGACCCCTTCGATCCCAAGCTTATATGGTCAAGAACTACCCGACATACAGCCAAACAATAACTAACACTGCAACTGACCGTGCAAGCATAACCACCACGGATTATGAAGGCGGGGTACCAGCAAACCCACAAAGGACATCT

>MF596095.1

AATGACCCCATTGCAAATGCAGTGGAAAGTGCTGTTAGCGCGCTTGCTGACACCACAATATCCCGGGTGACCGCAGCCAACACTGCAGTTAGTACCCACTCCCTGGGCACAGGGCGTGTACCAGCATTGCAAGCTGCGGAAACGGGAGCAAGTTCTAATGCTAGTGATGAAAACCTTATTGAGACTCGCTGTGTGATGAATCGAAACGGGGTTAATGAGGCGAGTGTGGAGCACTTTTACTCTCGTGCAGGGCTGGTAGGAGTTGTGGAGGTAAAGGACTCGGGCACTAGCCTGGATGGGTACACAGTTTGGCCCATAGATGTGATGGGCTTCGTGCAGCAGCGGCGCAAACTAGAGCTGTCAACATACATGCGCTTTGATGCCGAGTTCACTTTTGTGTCCAACCTCAGTGATAGCACAACGCCCGGGATGCTGCTGCAGTATATGTATGTACCACCAGGGGCCCCTAAGCCGGATAGCAGGAAATCGTACCAATGGCAGACCGCTACTAACCCGTCGATATTCGCAAAATTGAGTGATCCACCCCCCCAGGTGTCTGTTCCGTTCATGTCGCCAGCAACAGCTTATCAGTGGTTTTATGATGGCTACCCCACATTTGGTGAACACAAACAAGCTACCAATTTGCAATATGGGCAGTGCCCTAACAACATGATGGGCCATTTTGCCATTCGAACTGTCAGTGAATCTACCACCGGGAAAAATATCCACGTTCGGGTGTACATGAGAATTAAGCACGTGAGAGCTTGGGTACCTAGACCCCTTCGATCCCAAGCTTATATGGTCAAGAACTACCCGACATACAGCCAAACAATAACTAACACTGCAACCGACCGAGCAAGCATAACCACCACGGATTATGAAGGCGGGGTACCAGCAAACCCACAAAGAACATCT

>MF596120.1

AATGATCCCATTACAAATGCAGTAGAAAGCGCTGTGAGCGCACTTGCTGACACCACAATATCCCGAGTGACCGCAGCCAACACTGCAGCTAGCACCCACTCCCTGGGAACAGGGCGCGTACCAGCATTGCAAGCGGCAGAAACGGGAGCAAGCTCTAATGCTAGTGATGAGAACCTTATTGAGACTCGCTGTGTGATGAATCGAAACGGGGTTAATGAGGCGAGTGTGGAACACTTTTACTCTCGTGCAGGGCTGGTAGGAGTTGTGGAGGTGAAGGACTCGGGCACTAGCCTGGATGGGTACACAGTTTGGCCCATAGATGTGATGGGCTTCGTGCAACAGCGGCGCAAGCTAGAGCTGTCAACATACATGCGCTTTGATGCTGAGTTCACTTTTGTGTCCAACCTCAATGACAGTACGACGCCCGGGATGCTGCTGCAGTATATGTATGTACCACCAGGGGCCCCTAAGCCGGATAGCAGGAAATCATACCAATGGCAGACTGCTACTAACCCATCGGTATTCGCAAAATTGAGTGATCCACCCCCCCAGGTATCTGTTCCGTTCATGTCGCCAGCAACAGCTTATCAGTGGTTTTATGATGGTTATCCTACATTTGGTGAACACAAGCAAGCTACCAATTTGCAATATGGGCAGTGTCCTAATAACATGATGGGCCATTTTGCCATTCGAACAGTCAGTGAAGCCACCACCGGGAAAAACGTCCACGTTCGGGTGTTCATGAGAATTAAGCACGTGAGAGCTTGGGTACCTAGACCCCTCCGATCCCAAGCTTATATGGTCAAGAATTACCCGACATACAGTCAAACAATAACTAACACTGCAACCGACCGTGCAAGCATAACCACCACGGATTATGAAGGCGGGGTACCAGCAAACCCACAAAGGACATCT

>MF596144.1

AATGACCCCATTACAAATGCAGTGGAAAGCGCTGTGAGCGCGCTTGCTGACACCACGATATCCCGGGTGACCGCAGCCAACACTGCAGCTAGCACCCACTCCCTAGGAACAGGGCGTGTACCAGCATTGCAAGCCGCAGAAACGGGAGCAAGCTCTAATGCTAGCGACGAGAACCTTATTGAGACTCGCTGTGTGATGAATCGAAACGGGGTTAATGAAGCGAGTGTGGAACACTTTTACTCTCGTGCAGGGCTGGTAGGAGTTGTGGAGGTGAAGGACTCGGGCACTAGCCTGGATGGGTACACAGTTTGGCCCATAGATGTGATGGGCTTCGTGCAACAGCGGCGCAAGCTAGAGCTGTCAACATACATGCGCTTTGATGCCGAGTTCACTTTTGTGTCCAACCTCAATGACAGCACGACGCCCGGGATGCTGCTGCAGTATATGTATGTACCACCAGGTGCCCCTAAGCCGGATAGTAGGAAATCATATCAATGGCAGACTGCTACTAACCCGTCGGTATTCGCAAAATTGAGTGATCCACCCCCCCAGGTATCTGTTCCGTTCATGTCGCCAGCAACAGCTTACCAGTGGTTTTATGATGGTTACCCTACATTTGGTGAGCACAAACAAGCTGCCAATTTGCAATATGGGCAGTGTCCTAATAACATGATGGGCCATTTTGCCATCCGAACAGTCAGTGAATCTACCACCGGGAAAAACGTCCACATTCGGGTGTTTATGAGAATTAAGCACGTGAGAGCTTGGGTGCCTAGACCCCTTCGATCCCAAGCTTATATGGTCAAGAACTACCCGACATACAGTCAAACAATAACTAACACTGCAACTGACCGTGCAAGCATAACCACCACGGATTATGAAGGCGGGGTACCAGCAAACCCACAAAGGACATCT

>MF962674.1

AATGATCCCATTGCAAATGCAGTGGAAAGTGCTGTGAGCGCGCTTGCTGACACCACAATATCTCGAGTGACCGCAGCCAGCACTGCAGCTAGCACCCACTCCCTGGGTACGGGGCGTGTACCAGCATTGCAAGCTGCAGAAACAGGAGCAAGTTCTAATGCTAGTGATGAAAACCTCATTGAGACTCGCTGTGTGATGAATCGAAACGGGGTCAATGAGGCGAGTGTGGAGCACTTTTACTCTCGTGCAGGGCTGGTAGGGGTTGTGGAGGTAAAGGACTCGGGCACTAGCTTGGATGGGTACACAGTTTGGCCCATAGACGTGATGGGCTTTGTGCAGCAGCGGCGCAAACTAGAGCTGTCAACATACATGCGCTTTGATGCCGAGTTCACTTTTGTGTCCAACCTCAATGATAGCACGACGCCCGGGATGCTGCTGCAGTATATGTATGTACCACCAGGGGCCCCTAAACCGGATAGTAGGAAATCATACCAATGGCAGACTGCTACTAACCCGTCGGTATTCGCAAAATTGAGTGATCCACCCCCCCAGGTGTCTGTCCCGTTCATGTCGCCAGCAACAGCTTATCAGTGGTTTTATGATGGCTACCCCACATTTGGTGAGCACAAACAAGCTACTAATTTGCAATATGGGCAGTGTCCTAACAACATGATGGGCCACTTTGCCATCCGAACAGTCAGCGAATCTACCACCGGGAAAAACGTCCACGTTCGGGTGTACATGAGAATTAAGCATGTGAGAGCTTGGGTACCCAGGCCCCTTCGGTCCCAAGCTTATATGGTCAAAAATTACCCGACATATAGCCAAACAATAACTAACACTGCAACCGACCGTGCAAGCATAACCACCACTGATTATGAAGGCGGGGTACCAGCAAACCCACAAAGAACATCT

>MF991297.1

AATGACCCCATTGCAAATGCAGTGGAAAGTGCTGTTAGCGCGCTTGCTGACACCACAATATCCCGGGTGACCGCAGCCAACACTGCAGTTAGTACCCACTCCCTGGGCACAGGGCGTGTACCAGCATTGCAAGCTGCGGAAACGGGAGCAAGTTCTAATGCTAGTGATGAGAACCTTATTGAGACTCGCTGTGTGATGAATCGAAACGGGGTTAATGAGGCGAGTGTGGAGCACTTTTACTCTCGTGCAGGGCTGGTAGGAGTTGTGGAGGTGAAGGACTCGGGCACTAGCCTGGATGGGTACACAGTTTGGCCCATAGATGTGATGGGCTTCGTGCAGCAGCGGCGCAAACTAGAGCTGTCAACATACATGCGCTTTGATGCCGAGTTCACTTTTGTGTCCAACCTCAGTGATAGCACAACGCCCGGGATGCTGCTGCAGTATATGTATGTACCACCAGGGGCCCCTAAGCCGGATAGCAGGAAATCATACCAATGGCAGACTGCTACTAACCCGTCGGTATTCGCAAAATTGAGTGATCCACCCCCCCAGGTGTCTGTTCCGTTCATGTCGCCAGCAACAGCTTATCAGTGGTTTTATGATGGCTACCCCACATTTGGTGAGCACAAACAAGCTACCAATTTGCAATATGGGCAGTGCCCTAACAACATGATGGGCCATTTTGCCATCCGAACTGTCAGTGAATCTACCACCGGGAAAAACATCCACGTTCGGGTGTACATGAGAATTAAGCACGTGAGAGCTTGGGTACCTAGACCCCTTCGATCCCAAGCTTATATGGTCAAGAATTACCCGACATACAGCCAAACAATAACTAACACTGCAACCGACCGTGCAAGCATAACCACCACGGATTACGAAGGCGGGGTACCAGCAAACCCACAAAGAACATCT

>MG018992.1

AATGACCCCATTGCAAATGCAGTGGAAAGTGCTGTGAGCGCGCTTGCTGACACCACAATATCCCGGGTGACCGCAGCCAACACTGCAGTTAGTACCCACTCCCTGGGCACAGGGCGTGTACCAGCATTGCAAGCTGCAGAAACGGGAGCAAGTTCTAATGCTAGTGATGAGAACCTTATTGAGACTCGCTGTGTGATGAATCGAAACGGGGTTAATGAGGCGAGTGTGGAACACTTTTACTCTCGTGCAGGGCTGGTAGGAGTTGTGGAGGTGAAGGACTCGGGCACTAGCCTGGATGGGTACACAGTTTGGCCCATAGATGTGATGGGCTTCGTGCAGCAGCGGCGCAAACTAGAGCTGTCAACATACATGCGCTTTGATGCCGAGTTCACTTTTGTGTCCAACCTCAGTGATAGCACAACGCCCGGGATGCTGCTGCAGTATATGTATGTACCACCAGGGGCCCCTAAGCCGGATAGCAGGAAATCATACCAATGGCAGACTGCTACTAACCCGTCGGTATTTGCAAAATTGAGTGATCCACCCCCCCAGGTGTCTGTTCCGTTCATGTCGCCAGCAACAGCTTATCAGTGGTTTTATGATGGTTACCCCACATTTGGTGAGCATAAACAAGCTACCAATTTGCAATATGGGCAGTGCCCTAACAACATGATGGGCCATTTTGCCATCCGAACTGTCAGTGAATCTACCACCGGGAAAAACATCCACGTTCGGGTGTACATGAGAATTAAGCACGTGAGAGCTTGGGTACCTAGACCCCTTCGATCCCAAGCTTATATGGTCAAGAATTACCCGACATACAGCCAAACAATAACTAACACTGCAACCGACCGTGCAAGCATAACCACCACGGATTATGAAGGTGGGGTACCAGCAAACCCACAAAGAACATCT

>MG252915.1

AATGATCCCATTACAAATGCAGTGGAAAGCGCTGTGAGCGCGCTCGCTGACACCACAATATCCCGGGTGACCGCAGCTAGCACTGCAGTTAGCACCCACTCCCTGGGAACAGGGCGTGTACCAGCATTGCAAGCAGCAGACACGGGAGCAAGCTCTAATGCTAGTGATGAGAACCTTATTGAGACTCGCTGTGTGATGAATCGAAACGGGGTTAATGAGGCGAGTGTGGAACACTTTTACTCTCGTGCAGGACTGGTAGGAGTTGTGGAGGTGAAGGACTCGGGCACTAGCCTGGATGGGTACACAGTTTGGCCCATAGATGTGATGGGCTTCGTGCAACAGCGGCGCAAGCTAGAGCTGTCAACATACATGCGCTTTGATGCCGAGTTCACTTTTGTGTCCAACCTTAATGACAGCACGACGCCCGGGATGCTGCTACAGTATATGTATGTACCACCAGGGGCCCCTAAGCCGGATAGCAGGAAATCATACCAATGGCAGACTGCTACTAACCCGTCAGTGTTTGCGAAATTGAGTGATCCACCCCCCCAGGTATCTGTCCCGTTCATGTCGCCAGCAACAGCTTATCAGTGGTTTTATGATGGTTACCCTACGTTTGGTGAGCACAAACAAGCCACCAATTTGCAATATGGGCAGTGTCCTAATAACATGATGGGCCATTTTGCCATCCGTACAGTCAGTGAATCTACCACCGGGAAAAACGTCCACGTTCGGGTGTTCATGAGAATTAAGCATGTGAGAGCTTGGGTACCCAGACCCCTCCGATCCCAAGCTTATATGGTCAAGAACTACCCGACATACAGCCAAACAATAACTAACACTGCAACTGACCGTGCAAGCATAACCACCACGGATTATGAAGGCGGGGTACCAGCAAACCCACAAAGGACATCT

>MG385755.1

AATGATCCCATTACAAATGCAGTGGAAAGCGCTGTGAGCGCGCTTGCCGACACCACTATATCCCGGGTGACCGCAGCCAACACTGCAGCCAGCACCCACTCCCTGGGGACAGGGCGTGTACCAGCATTGCAAGCCGCAGAAACGGGAGCAAGCTCTAATGCTAGTGATGAGAACCTTATTGAGACTCGCTGTGTGATGAATCGAAACGGGGTTAATGAGGCGAGTGTGGAACACTTTTACTCTCGTGCAGGTCTGGTAGGAGTTGTGGAGGTGAAGGACTCGGGCACTAGTCTGGATGGGTACACAGTTTGGCCCATAGATGTGATGGGATTCGTGCAGCAGCGGCGCAAGCTAGAGCTGTCAACATACATGCGCTTTGATGCCGAGTTCACTTTTGTATCCAACCTCAATGACAGCACGACGCCCGGGATGCTGCTGCAGTATATGTATGTACCACCAGGGGCCCCTAAGCCGGATAGCAGGAAATCATATCAATGGCAGACTGCTACTAACCCGTCGATATTCGCAAAATTGAGTGATCCACCTCCCCAGGTATCTGTTCCGTTCATGTCGCCAGCAACAGCTTATCAGTGGTTTTATGATGGCTACCCCACATTTGGTGAGCACAAACAAGCTACCAACTTGCAATATGGGCAGTGTCCTAATAACATGATGGGCCATTTTGCTATCCGAACAGTCAGTGAATCTACCACCGGGAAAAACGTCCACGTTCGGGTGTACATGAGAATTAAGCACGTGAGAGCTTGGGTGCCTAGACCCCTTCGATCCCAAGCTTATATGGTCAAGAACTACCCGACATACAGCCAAACAATAACTAACACTGCAACTGACCGTGCAAGCATAACTACCACGGATTATGAAGGCGGGGTACCAGCAAACCCACAAAGGACATCT

>MG488226.1

AATGACCCCATTGCAAATGCAGTGGAAAGTGCTGTTAGCGCGCTTGCTGACACCACAATATCCCGGGTGACCGCAGCCAACACTGCAGTTAGTACCCACTCCCTGGGCACAGGGCGTGTACCAGCATTGCAAGCTGCGGAAACGGGAGCAAGTTCTAATGCTAGTGATGAGAACCTTATTGAGACTCGCTGTGTGATGAATCGAAACGGGGTTAATGAGGCGAGTGTGGAGCACTTTTACTCTCGTGCAGGGCTGGTAGGAGTTGTGGAAGTGAAGGACTCGGGCACTAGCCTGGATGGGTACACAGTTTGGCCCATAGACGTGATGGGCTTCGTGCAGCAGCGGCGCAAACTAGAGCTGTCAACATACATGCGCTTTGATGCCGAGTTCACTTTTGTGTCCAACCTCAGTGATAGCACAACGCCCGGGATGCTGCTGCAGTATATGTATGTACCACCAGGGGCCCCTAAGCCGGATAGCAGGAAATCATACCAATGGCAGACTGCTACTAACCCATCGGTATTCGCAAAATTGAGTGATCCACCCCCTCAGGTGTCTGTTCCGTTCATGTCGCCAGCAACAGCTTATCAGTGGTTCTATGATGGCTACCCCACATTTGGTGAGCACAAACAAGCTACCAATTTGCAATATGGGCAGTGCCCTAACAACATGATGGGCCATTTTGCCATCCGAACTGTTAGTGAATCTACCACCGGGAAAAACATCCACGTTCGGGTGTACATGAGGATTAAGCACGTGAGAGCTTGGGTACCTAGACCCCTTAGATCCCAAGCTTATAAGGTCAAGAATTTCCCGACATACAGCCAAACAATTCCTATCTCTGCAACCGTTCGTGCAAGCATAACCACCACGGACTATGAAGGCGGGGTACCAGCAAACCCACAAAGAACATCT

>MH018472.1

AATGACCCCATTGCAAATGCAGTGGAAAGTGCTGTTAGCGCGCTTGCTGACACCACAATATCCCGGGTGACCGCAGCGAACACTGCAGTTAGTACCCACTCCCTGGGCACAGGGCGTGTACCAGCATTGCAAGCTGCGGAAACGGGAGCAAGTTCTAATGCTAGTGATGAGAACCTTATTGAGACTCGCTGTGTGATGAACCGAAACGGGGTTAATGAGGCGAGTGTGGAACACTTTTACTCTCGTGCAGGGCTGGTAGGAGTTGTGGAGGTGAAGGACTCGGGCACTAGCCTGGATGGGTATACAGTTTGGCCCATAGATGTGATGGGCTTCGTGCAGCAGCGGCGCAAACTAGAGCTGTCAACATACATGCGCTTTGATGCCGAGTTCACTTTTGTGTCCAACCTCAGTGATAGCACAACGCCCGGGATGCTGCTGCAGTATATGTATGTACCACCAGGGGCCCCTAAGCCGGATAGCAGGAAATCATACCAATGGCAGACTGCTACTAACCCGTCAGTATTCGCAAAATTGAGTGATCCACCCCCCCAGGTGTCTGTTCCGTTCATGTCGCCAGCAACAGCTTATCAGTGGTTTTATGATGGTTACCCCACATTTGGTGAGCACAAACAAGCTACCAATTTGCAATATGGGCAGTGCCCTAACAACATGATGGGCCATTTTGCCATCCGAACTGTCAGTGAATCTACCACCGGGAAAAACATCCACGTTCGGGTGTACATGAGAATTAAGCACGTGAGAGCTTGGGTACCTAGACCCCTTCGATCCCAAGCTTATATGGTCAAGAATTACCCGACATACAGCCAAACAATAACTAACGCTGCAACCGACCGTGCAAGCATAACCACCACGGATTATGAAGGCGGGGTACCAGCAAACCCGCAAAGAACATCT

>MH018476.1

AATGACCCCATTGCAAATGCAGTGGAAAATGCTGTGAGTGCGCTTGCTGACACCACAATATCCCGGGTGACCGCAGCCAACACTGCAGTTAGTACCCACTCCCTGGGCACAGGGCGTGTACCAGCATTGCAAGCTGCAGAAACGGGAGCAAGTTCTAATGCTAGTGATGAGAACCTTATTGAGACTCGCTGTGTGATGAATCGAAACGGGGTTAATGAGGCGAGTGTGGAACACTTTTACTCTCGTGCGGGGCTGGTAGGAGTTGTGGAGGTGAAGGATTCGGGCACTAGCCTGGATGGGTACACAGTTTGGCCCGTAGATGTGATGGGCTTCGTGCAGCAGCGGCGCAAACTAGAGCTGTCAACATACATGCGCTTTGATGCCGAGTTCACTTTCGTGTCCAACCTCAATGATAGCACAACGCCCGGAATGTTGCTGCAGTATATGTATGTACCACCAGGGGCCCCTAAGCCGGATAGCAGGAAATCATACCAATGGCAGACTGCTACTAACCCGTCGGTATTTGCGAAATTGAGTGATCCACCCCCCCAGGTGTCTGTTCCGTTCATGTCGCCAGCAACAGCTTATCAGTGGTTTTATGATGGTTACCCCACATTTGGTGAGCACAAACAAGCTACCAATTTGCAATATGGGCAGTGTCCTAACAACATGATGGGCCATTTTGCCATCCGAACTGTTAGTGAGTCTACCACCGGGAAAAACGTCCACGTTCGGGTGTACATGAGGATTAAGCACGTGAGAGCTTGGGTACCTAGACCCCTTCGATCCCAAGCTTATATGGTGAAGAATTACCCGACATACAGCCAAACAATAACTAACACTGCAACCGACCGTGCAAGCATAACCACCACGGATTATGAAGGCGGGATACCAGCAAATCCACAAAGAACATCT

>MH018486.1

AATGATCCCATTACAAATGCAGTAGAAAGCGCTGTGAGCGCGCTTGCTGACACCACAATATCCCGAGTGACCGCAGCCAACACTGCAGCTAGCACCCACTCACTGGGAACAGGGCGCGTACCAGCATTGCAAGCGGCAGAAACGGGAGCAAGCTCTAATGCTAGTGATGAGAACCTTATTGAGACTCGCTGTGTGATGAATCGAAACGGGGTTAATGAGGCGAGTGTGGAACACTTTTACTCTCGTGCAGGGCTGGTAGGAGTTGTGGAGGTGAAGGACTCGGGCACTAGCCTGGATGGGTACACAGTTTGGCCCATAGATGTGATGGGCTTCGTGCAACAGCGGCGCAAGCTAGAGCTGTCAACATACATGCGCTTTGATGCTGAGTTCACTTTTGTGTCCAACCTCAATGACAGTACGACGCCCGGGATGCTGCTGCAGTATATGTATGTACCACCAGGGGCCCCTAAGCCGGATAGCAGGAAATCATACCAATGGCAGACTGCTACTAACCCGTCGGTATTCGCAAAATTGAGTGATCCACCCCCCCAGGTATCTGTTCCGTTCATGTCGCCAGCAACAGCTTATCAGTGGTTTTATGATGGTTACCCTACATTTGGTGAACACAAGCAAGCTACCAATTTGCAATATGGGCAGTGTCCTAATAACATGATGGGCCATTTTGCCATTCGAACAGTCAGTGAAGCCACCACCGGGAAAAACGTCCATGTTCGGGTGTTCATGAGAATTAAGCACGTGAGAGCTTGGATACCTAGACCCCTCCGATCCCAAGCTTATATGGTCAAGAATTACCCGACATACAGCCAAACAATAACTAACACTGCAACCGACCGTGCAAGCATAACCACCACGGATTATGAAGGCGGGGTACCAGCAAACCCACAAAGGACATCT

>MH018507.1

AATGATCCCATTACAAATGCAGTGGAAAGCGCTGTGAGCGCGCTTGCTGACACCACAATATCCCGGGTGACCGCAGCCAACACTGCAGCTAGCACCCACTCCCTGGGAACAGGGCGTGTACCAGCATTGCAAGCCGCAGAAACGGGAGCAAGCTCTAATGCCAGTGATGAGAACCTTATTGAGACCCGCTGTGTGATGAATCGAAACGGGGTTAATGAGGCGAGTGTGGAACACTTTTACTCTCGTGCAGGGCTGGTAGGAGTTGTGGAGGTGAAGGACTCGGGCACTAGCCTGGATGGGTACACAGTTTGGCCCATAGATGTGATGGGCTTCGTGCAACAGCGGCGCAAGCTAGAGTTGTCAACATACATGCGCTTTGATGCCGAGTTCACTTTTGTGTCCAACCTCAATGACAGCACGACGCCTGGCATGCTGCTGCAGTATATGTATGTGCCACCAGGGGCCCCTAAGCCAGATAGCAGGAAATCATACCAATGGCAGACTGCTACTAACCCGTCGATATTCGCAAAATTGAGTGATCCACCCCCCCAGGTATCTGTCCCGTTCATGTCGCCAGCAACGGCTTATCAGTGGTTTTATGATGGTTACCCTACATTTGGTGAACACAAACAAGCCACCAATTTGCAATATGGGCAATGTCCTAATAACATGATGGGCCATTTTGCTATCCGAACAGTCAGTGAGTCTACCACCGGGAAAAACGTCCACGTTCGGGTGTACATGAGAATTAAGCACGTGAGAGCTTGGGTACCTAGACCCCTTCGATCCCAAGCATATATGGTCAAGAACTACCCGACATACAGCCAAACAATAACTAACACTGCAGCTGACCGTGCAAGCATAACCACCACGGATTATGAAGGCGGGGTACCAGCAAACCCACAGAGGACATCT

>MH018535.1

AATGATCCCATTACAAATGCAGTGGAAAGCGCTGTGAGCGCGCTTGCTGACACCACAATATCCCGGGTGACCGCAGCCAACACTGCAGCTAGCACCCACTCCCTGGGAACAGGGCGTGTACCAGCATTGCAAGCCGCAGAAACGGGAGCAAGCTCTAATGCCAGTGATGAGAACCTTATTGAGACCCGCTGTGTGATGAATCGAAACGGGGTTAATGAGGCGAGTGTGGAACACTTTTACTCTCGTGCAGGGCTGGTAGGAGTTGTGGAGGTGAAGGACTCGGGCACTAGCCTGGATGGGTACACAGTTTGGCCCATAGATGTGATGGGCTTCGTGCAGCAGCGGCGCAAGCTAGAGTTATCAACATACATGCGCTTTGATGCCGAGTTCACTTTTGTGTCCAACCTCAATGACAGCACGACACCCGGGATGCTGCTGCAGTACATGTATGTGCCACCGGGGGCCCCTAAGCCAGATAGCAGGAAATCATACCAATGGCAGACTGCTACTAACCCGTCGATATTCGCAAAATTGAGTGATCCACCCCCCCAGGTATCTGTCCCGTTCATGTCGCCAGCAACGGCTTATCAGTGGTTTTATGATGGTTACCCTACATTTGGTGAACACAAACAAGCCACCAATTTGCAATATGGGCAATGTCCTAATAACATGATGGGCCATTTTGCTATCCGAACAGTCAGTGAATCTACCACCGGGAAAAACGTCCACGTTCGGGTGTACATGAGAATTAAGCACGTGAGAGCTTGGGTACCTAGACCCCTTCGATCCCAAGCATATATGGTCAAGAACTACCCGACATACAGCCAAACAATAACTAACACTGCAGCTGACCGTGCAAGCATAACCACCACGGATTATGAAGGTGGGGTACCAGCAAACCCGCAGAGGACATCT

>MH086137.1

AATGACCCTATTGCAAATGCAGTGGAAAGTGCTGTTAGCGCGCTTGCTGACACCACAATATCCCGGGTGACCGCAGCCAACACTGCAGTTAGTACCCACTCCCTGGGCACAGGGCGTGTACCAGCATTGCAAGCTGCGGAAACGGGAGCAAGTTCTAATGCTAGTGATGAGAACCTTATTGAGACTCGCTGTGTGATGAATCGAAACGGGGTTAATGAGGCGAGTGTGGAGCACTTTTACTCTCGTGCAGGGCTGGTAGGAGTTGTGGAGGTAAAGGACTCGGGCACTAGCCTGGATGGGTACACAGTTTGGCCCATAGATGTGATGGGCTTCGTGCAGCAGCGGCGCAAACTAGAGCTGTCAACATACATGCGCTTTGATGCCGAGTTCACTTTTGTGTCCAACCTCAGTGATAGCACAACGCCCGGGATGCTGTTGCAGTATATGTATGTACCACCAGGGGCCCCTAAGCCGGATAGCAGGAAATCGTACCAATGGCAGACTGCTACTAACCCGTCGGTATTCGCAAAATTGAGTGATCCACCCCCCCAGGTGTCTGTTCCGTTCATGTCGCCAGCAACAGCTTATCAGTGGTTTTATGATGGCTACCCCACATTTGGTGAGCACAAACAAGCTACCAATTTGCAATATGGGCAGTGCCCTAACAACATGATGGGCCATTTTGCCATTCGAACTGTCAGTGAATCTACCACCGGGAAAAACATCCACGTTCGGGTGTACATGAGAATTAAGCACGTGAGAGCTTGGGTACCTAGACCCCTTCGATCCCAAGCTTATATGGTCAAGAACTACCCGACATACAGTCAAACAATAACTAACACTGCAACCGACCGTGCAAGCATAACCACCACGGATTATGAAGGCGGGGTACCAGCAAACCCACAAAGAACATCT

>MH086142.1

AATGACCCCATTGCAAGTGCAGTGGAAAGTGCTGTTAGCGCGCTTGCTGACACCACAATATCCCGGGTGACCGCAGCCAACACTGCAGTTAGTACCCACTCCCTGGGCACAGGGCGTGTACCAGCATTGCAAGCTGCGGAAACGGGAGCAAGTTCTAATGCTAGTGATGAGAACCTCATTGAGACTCGCTGTGTGATGAATCGAAACGGGGTTAATGAGGCGAGTGTGGAGCACTTTTACTCTCGTGCAGGGCTGGTAGGAGTTGTGGAGGTGAAGGACTCGGGCACTAGCCTGGATGGGTACACAGTTTGGCCCATAGATGTGATGGGCTTCGTGCAGCAGCGGCGCAAACTAGAGCTGTCAACATACATGCGCTTTGATGCCGAGTTCACTTTTGTGTCCAACCTCAGTGATAGCACAACGCCCGGGATGCTGCTGCAGTACATGTATGTACCACCAGGGGCCCCTAAGCCGGATAGCAGGAAATCATACCAATGGCAGACTGCTACTAACCCGTCGGTATTCGCAAAATTGAGTGATCCACCCCCCCAGGTGTCTGTTCCGTTCATGTCGCCAGCAACAGCTTATCAGTGGTTTTATGATGGCTACCCCACATTTGGTGAGCACAAACAAGCTACCAATTTGCAATATGGGCAGTGCCCTAACAACATGATGGGCCATTTTGCCATCCGAACTGTCAGTGAATCTACCACCGGGAAAAACATCCATGTTCGGGTGTACATGAGAATTAAGCACGTGAGAGCTTGGGTACCTAGACCCCTTCGATCCCAAGCTTATATGGTCAAGAATTACCCGACATACAGCCAAACAATAACCAACACTGCAACCGATCGTGCAAGCATAACCACCACGGATTATGAAGGCGGGGTACCAGCAAACCCACAAAGAACATCT

>MH086148.1

AATGATCCCATTACAAATGCGGTGGAAAGCGCTGTGAGCGCGCTTGCTGACACCACAATATCCCGGGTAACCGCAGCCAACACTGCAGCTAGCACCCACTCCCTGGGAACAGGGCGTGTACCAGCATTGCAAGCCGCAGAAACGGGAGCAAGCTCTAATGCTAGTGATGAGAACCTTATTGAGACCCGCTGTGTGATGAATCGAAACGGGGTTAATGAGGCGAGCGTGGAACACTTTTACTCTCGTGCAGGGCTGGTAGGAGTTGTGGAGGTGAAGGACTCGGGCACTAGCCTGGATGGGTACACAGTCTGGCCCATAGATGTGATGGGCTTCGTGCAACAGCGGCGCAAGCTAGAGCTATCAACATACATGCGCTTTGATGCCGAGTTCACTTTTGTGTCCAACCTCAATGACAGCACAACACCCGGGATGCTGCTGCAGTATATGTATGTACCACCAGGGGCCCCTAAGCCGGATAGCAGGAAATCATATCAATGGCAGACTGCTACTAACCCGTCGATATTCGCAAAGTTGAGTGATCCACCCCCCCAGGTATCTGTTCCGTTCATGTCGCCAGCAACAGCTTATCAGTGGTTTTATGATGGTTACCCTACATTTGGTGAGCACAAACAAGCTACCAATTTGCAATATGGGCAGTGTCCTAATAACATGATGGGCCATTTTGCCATCCGAACAGTCAGTGAATCTACCACCGGGAAAGACGTCCGCGTTCGGGTGTACATGAGAATTAAGCACGTGAGAGCTTGGGTACCTAGACCCCTTCGATCCCAAGCTTATATGGTCAAGAACTACCCGACATACAGCCAAACAATAACTAACACTGCAGCTGACCGTGCAAGCATAACCACCACGGATTATGAAGGCGGGGTACCAGCAAACCCACAAAGGACATCT

>MH086162.1

AATGACCCCATTACAAATGCAGTGGAAAGCGCTGTGAGCGCGCTTGCTGACACCACAATATCCCGGGTGACTGCAGCCAACACCGCAGCTAGCACCCACTCCCTGGGAACAGGGCGTGTACCAGCATTACAAGCGGCAGAAACGGGAGCAAGCTCTAATGCTAGTGATGAGAACCTTATTGAGACTCGCTGTGTGATGAATCGAAACGGGGTTAATGAGGCGAGTGTGGAGCATTTTTACTCTCGTGCAGGGCTGGTAGGAGTTGTGGAGGTGAAGGACTCGGGCACTAGCCTGGATGGGTACACAGTTTGGCCCATAGATGTGATGGGCTTCGTGCAACAGCGGCGCAAGCTAGAGCTGTCAACATACATGCGCTTTGATGCTGAGTTCACTTTTGTGTCCAACCTCAATGACAGCACGACGCCCGGGATGCTACTGCAGTATATGTATGTACCACCGGGGGCCCCTAAACCGGATAGCAGGAAATCATATCAATGGCAGACTGCTACTAACCCGTCGGTATTCGCGAAATTGAGTGATCCACCCCCCCAGGTATCTGTTCCGTTCATGTCGCCAGCAACAGCTTATCAGTGGTTTTATGATGGTTACCCTACATTTGGTGAGCACAAACAAGCTACCAACTTGCAATATGGGCAGTGTCCTAATAACATGATGGGCCATTTTGCCATCCGAACAGTCAGTGAAGCTACCACCGGGAAAAACGTCCACGTTCGGGTGTACATGAGAATTAAGCACGTGAGAGCCTGGGTACCTAGACCCCTTCGATCCCAAGCTTATATGGTCAAGAACTACCCGACATACAGCCAACCAAGAACTAACACTGCAACTGACCGTGCAAGCATAACCACCACGGATTATGAAGGCGGGGTACCAGCAAACCCACAAAGGACATCT

>MH086181.1

AATGATCCCATTACAAATGCAGTGGAAAGCGCTGTGAGCGCGCTTGCTGACACCACAATATCCCGGGTGACCGCAGCCAACACTGCAGCTAGCACCCACTCCCTGGGAACAGGGCGTGTGCCAGCATTGCAAGCCGCAGAAACGGGAGCAAGCTCTAATGCCAGTGATGAGAACCTTATCGAGACTCGCTGTGTGATGAATCGAAACGGGGTTAATGAGGCGAGTGTGGAACACTTTTACTCTCGTGCAGGGCTGGTAGGAGTTGTGGAGGTGAAGGACTCGGGCACTAACCTGGATGGGTACACAGTTTGGCCTGTAGATGTGATGGGCTTCGTGCAACAGCGACGCAAGCTAGAGCTGTCAACATACATGCGCTTTGATGCCGAGTTCACTTTTGTGTCCAACCTTAATGATAGCACGACGCCCGGGATGCTGCTGCAGTATATGTATGTACCACCAGGGGCTCCTAAGCCGGATGGCAGGAAATCATATCAATGGCAGACTGCTACTAACCCGTCGGTATTCGCAAAATTGAGTGATCCACCCCCCCAGGTATCTGTCCCGTTCATGTCACCAGCAACAGCTTATCAGTGGTTTTATGATGGTTACCCTACATTTGGTGAGCACAAACAAGCTACCAATTTGCAATATGGGCAGTGTCCTAATAACATGATGGGCCATTTTGCCATCCGAACAGTCAGTGAATCTACCACCGGAAAAAACGTCCACGTTCGGGTGTACATGAGAATTAAGCACGTGAGAGCTTGGGTACCTAGACCCCTCCGATCCCAAGCTTATATGGTCAAGAACTACCCGACATACAGCCAAACAATAACTAACACTGCAACTGATCGTGCAAGTATAACCACCACGGATTATGAAGGCGGGGTACCAGCAAACCCACAAAGGACATCT

>MH111053.1

AATGATCCCATTACAAATGCAGTAGAAAGCGCTGTGAGCGCGCTTGCTGACACCACAGTATCCCGGGTGACCGCAGCCAACACTGCAGCTAGCACCCACTCCCTGGGAACAGGGCGTGTACCAGCGTTGCAAGCCGCAGAAACGGGAGCAAGCTCTAATGCTAGTGATGAGAACCTTATTGAGACCCGCTGTGTGATGAATCGAAACGGGGTTAATGAAGCGAGTGTGGAACACTTTTACTCTCGTGCAGGACTGGTAGGAGTTGTGGAGGTGAAGGACTCGGGCACTAGCCTGGATGGGTATACAGTTTGGCCCATAGATGTGATGGGCTTCGTGCAACAGCGGCGCAAGCTAGAACTGTCAACATACATGCGCTTTGATGCCGAGTTCACTTTTGTGTCCAACCTTAATGACAGCACGACGCCCGGGATGATGCTGCAGTATATGTATGTGCCACCAGGGGCCCCTAAGCCAGATAGCAGGAAATCATATCAATGGCAGACTGCTACTAACCCGTCGGTATTCGCAAAATTGAGTGATCCACCCCCCCAGGTATCTGTCCCGTTCATGTCGCCAGCAACGGCTTATCAGTGGTTTTATGATGGTTACCCTACATTTGGTGAGCACAAACAAGCTGCCAATTTGCAATATGGGCAGTGTCCTAATAACATGATGGGCCATTTTGCCATCCGAACAGTCAGTGAATCTACCACCGGGAAAAACGTCCACGTTCGGGTGTACATGAGAATTAAGCACGTGAGAGCTTGGGTACCTAGACCCCTTCGATCCCAAGCCTATATGGTCAAGAACTACCCGACATACAGCCAAACAATAACTAACACTGCAGCTGACCGTGCAAGCATAACCACCACGGATTATGAAGGCGGGGTACCAGCAAACCCACAAAGGACATCT

>MH371303.1

AATGATCCCATTACAAATGCCGTGGAAAGTGCTGTGAGCGCGCTTGCTGACACCACAATATCCCGGGTGACCGCAGCCAACACTGCAGTCAGCACCCACTCCCTGGGTACGGGGCGCGTACCAGCACTGCAAGCTGCAGAGACGGGAGCAAGTTCTAATGCTAGTGATGAGAACCTTATTGAGACTCGCTGTGTGATGAATCGAAACGGGGTCAATGAGGCGAGTGTGGAACACTTTTACTCTCGTGCAGGGCTGGTAGGGGTTGTGGAGGTGAAGGACTCGGGCACTAGCTTGGATGGGTACACAGTTTGGCCCATAGACGTGATGGGCTTTGTGCAACAGCGGCGCAAACTAGAACTGTCAACGTACATGCGCTTTGATGCCGAGTTCACTTTTGTGTCCAACCTCAGTGATAGCACGACACCCGGGATGCTACTGCAGTATATGTATGTACCACCAGGGGCCCCCAAGCCGGATAGCAGGAAATCATACCAATGGCAGACTGCTACTAACCCGTCAGTATTCGCAAAGTTAAGTGATCCACCCCCCCAAGTGTCTGTCCCGTTCATGTCGCCAGCAACAGCTTATCAGTGGTTTTATGATGGTTACCCTACATTTGGTGAACACAAACAAGCCACTAATTTACAGTATGGACAGTGCCCTAACAACATGATGGGCCATTTTGCCATCCGAACAGTCAGCGAATCTACCACCGGAAAAAACGTCCACGTTCGAGTGTACATGAGAATCAAGCACGTGAGAGCTTGGGTACCTAGACCCCTTCGGTCCCAAGCATATATGGTCAAGAATTACCCGACGTATAGCCAAACAATAACTAACACTGCAACCGACCGTGCAAGTATAACCACCACGGATTATGAAGGCGGGGTACCAGCAAACCCACAAAGAACATCT

>MH539784.1

AACGATCCCATTACAAGTGCAGTGGAAAGCGCTGTGAGCGCGCTTGCTGATACCACAATATCCCGGGTGACCGCAGCCAACACTGCAGTTAGCACCCATTCCCTGGGAACAGGGCGCGTACCAGCGCTGCAAGCTGCAGAAACGGGAGCAAGCTCTAATGCTAGTGATGAGAACCTTATTGAGACCCGCTGTGTGATGAATCGAAACGGGGTTAATGAAGCGAGTGTAGAACATTTTTACTCTCGTGCAGGGCTAGTAGGAGTTGTGGAGGTGAAGGACTCGGGTACTAGCCTAGATGGGTACACAGTTTGGCCCATAGATGTGATGGGCTTCGTGCAACAGCGGCGCAAACTAGAGCTATCAACATACATGCGCTTTGATGCCGAGTTCACTTTCGTGTCCAATCTCAGTGACAGCACGACACCCGGGATGCTGCTGCAGTATATGTATGTACCACCAGGGGCCCCTAAGCCGGATAGTAGGAAGTCATACCAATGGCAGACTGCTACTAACCCGTCGGTATTCGCAAAATTGAGTGATCCACCCCCCCAGGTGTCTGTCCCGTTTATGTCGCCAGCGACAGCTTATCAGTGGTTTTATGATGGTTACCCTACATTTGGTGAGCACAAACAGGCTACCAATTTGCAATATGGGCAGTGCCCTAACAACATGATGGGCCATTTTGCCATCCGAACAGTTAGTGAATCTACCACCGGGAAAAACGTCCACGTTCGGGTGTATATGAGAATTAAGCATGTGAGAGCTTGGGTACCTAGACCCCTTCGATCCCAAGCTTATATGGTTAAGAATTACCCGACATACAGCCAAACAATAACTAACACTGCAACCGACCGCGCAAGCATAACCACCACGGATTATGAAGGCGGGGTACCAGCAAACCCACAAAGGACATCT

>MH539786.1

AACGATCCCATTACAAGTGCAGTGGAAAGCGCTGTGAGCGCGCTTGCTGATACCACAATATCCCGGGTGACCGCAGCCAACACTGCAGTTAGCACCCACTCCCTGGGAACAGGGCGTGTACCAGCGCTGCAAGCTGCAGAAACGGGAGCAAGTTCTAATGCTAGTGATGAGAACCTTATTGAGACCCGCTGTGTGATGAATCGAAACGGGGTTAATGAAGCGAGTGTAGAACACTTTTACTCTCGTGCAGGGTTGGTAGGAGTTGTGGAGGTGAAGGACTCGGGTACTAGCCTAGATGGGTACACAGTTTGGCCCATAGATGTGATGGGCTTCGTGCAACAGCGGCGCAAACTAGAGCTATCAACATACATGCGCTTTGATGCCGAGTTCACTTTTGTGTCCAACCTCAGTGACAGCACGACACCCGGGATGCTGCTGCAGTATATGTATGTACCACCAGGGGCCCCTAAGCCGGATAGTAGGAAGTCATATCAATGGCAGACTGCTACTAACCCGTCGGTATTCGCAAAATTGAGTGATCCGCCCCCCCAGGTGTCTGTCCCGTTCATGTCGCCAGCAACAGCTTATCAGTGGTTTTATGATGGTTACCCTACATTTGGTGAGCATAAACAAGCTACCAATTTGCAATATGGGCAGTGTCCTAACAACATGATGGGCCATTTTGCCATCCGAACAGTTAGTGAATCTACCACCGGGAAAAACGTCCACGTTCGGGTGTATATGAGAATCAAGCACGTGAGAGCTTGGGTGCCTAGACCCCTTCGATCCCAGGCTTATATGGTCAAGAATTACCCGACATACAGCCAAACAATAACTAACACTGCAACCGACCGCGCAAGCATAACCACCACGGATTATGAAGGCGGGGTACCAGCAAACCCACAAAGGACATCT

>MH544970.1

AATGACCCCATTGCAAATGCAGTGGAAAGTGCTGTTAGCGCGCTTGCTGACACCACAATATCCCGGGTGACCGCAGCCAACACTGCAGTTAGTACCCACTCCCTGGGCACAGGGCGTGTACCAGCATTGCAAGCTGCGGAAACGGGAGCAAGTTCTAATGCTAGTGATGAGAACCTTATTGAGACTCGCTGTGTGATGAATCGAAACGGGGTTAATGAGGCGAGTGTGGAACACTTTTACTCTCGTGCAGGGCTGGTAGGAGTTGTGGAGGTGAAGGACTCGGGCACTAGCCTGGACGGGTACACAGTTTGGCCCATAGATGTGATGGGCTTCGTGCAGCAGCGGCGCAAACTAGAGCTGTCAACATACATGCGCTTTGATGCCGAGTTCACTTTTGTGTCCAACCTCAGTGATAGCACAACGCCCAGGATGCTGCTGCAGTATATGTATGTACCACCAGGGGCCCCTAAGCCGGATAGCAGGAAATCATACCAATGGCAGACTGCTACTAACCCGTCGGTATTCGCAAAATTGAGTGATCCACCCCCCCAGGTGTCTGTTCCGTTCATGTCGCCAGCAACAGCTTATCAGTGGTTTTATGATGGTTACCCCACATTTGGTGAGCACAAACAAGCTACCAATTTGCAATATGGGCAGTGCCCTAACAACATGATGGGCCATTTTGCCATCCGAACTGTCAGTGAATCTACCACCGGGAAAAACATCCACGTTCGGGTGTACATGAGAATTAAGCACGTGAGAGCTTGGGTACCTAGACCCCTTCGATCCCAAGCTTATATGGTCAAGAATTACCCGACATACAGCCAAACAATAACTAACACTGCAACCGACCGTGCAAGCATAACCACCACGGATTATGAAGGCGGGGTACCAGCAAACCCACAAAGAACATCT

>MH716169.1

AATGATCCCATTACAAATGCAGTGGAAAGCGCTGTGAGCGCGCTTGCTGACACCACAATATCCCGGGTGACCGCAGCCAATACTGCAGCTAGCACCCACTCCCTGGGAACAGGGCGTGTACCAGCACTGCAAGCCGCAGAAACGGGAGCAAGCTCTAATGCTAGTGATGAGAACCTTATTGAGACTCGCTGTGTGATGAATCGAAACGGGGTTAATGAGGCGAGTGTGGAACACTTCTACTCTCGTGCAGGGCTGGTAGGAGTTGTGGAGGTGAAGGACTCGGGCACTAGCTTGGATGGGTACACAGTTTGGCCCATAGATGTGATGGGCTTCGTGCAACAGCGGCGCAAGCTAGAACTGTCAACATACATGCGCTTTGATGCCGAGTTCACTTTTGTGTCCAACCTCAATGACAGCACGACGCCCGGGATGCTGTTGCAGTATATGTATGTACCACCAGGGGCCCCTAAGCCGGATAGCAGGAAATCGTATCAATGGCAGACTGCTACTAACCCGTCGGTATTCGCAAAATTGAGTGATCCACCCCCCCAGGTATCTGTCCCGTTCATGTCGCCAGCAACAGCTTATCAGTGGTTTTATGATGGTTACCCTACATTCGGTGAGCACAAACAAGCTACCAATTTGCAATACGGGCAGTGTCCTAACAACATGATGGGCCATTTCGCCATCCGAACAGTCAGTGAATCCACCACCGGGAAAAACGTCCACGTTCGGGTGTACATGAGAATTAAGCACGTGAGAGCTTGGGTACCTAGACCCCTTCGATCCCAAGCTTATATGGTTAAGAACTACCCGACATACAGCCAAACAATAACTAACACTGCAACTGACCGTGCAAGCATAACCACCACGGATTATGAAGGCGGGGTGCCAGCAAACCCACAAAGGACATCT

>MK167090.1

AACGATCCCATTACAAATGCAGTGGAAAGCGCTGTGAGCGCGCTTGCTGACACCACAATATCCCGGGTGACCGCAGCCAACACTGCAGCTAGCACCCACTCCCTGGGAACAGGGCGTGTACCAGCATTGCAAGCCGCAGAAACGGGAGCAAGCTCTAATGCTAGTGATGAGAACCTAATTGAGACCCGCTGTGTGATGAATCGAAACGGGGTTAATGAGGCGAGTGTGGAACACTTTTACTCTCGTGCAGGGCTGGTAGGGGTTGTGGAGGTGAAAGACTCGGGCACTAGCCTGGATGGGTACACAGTTTGGCCCATAGATGTGATGGGCTTTGTACAACAGCGACGCAAGTTAGAGCTGTCAACATACATGCGCTTCGATGCTGAGTTCACTTTTGTGTCCAACCTCAATGACAGCACGACGCCCGGGATGCTGCTGCAGTATATGTATGTACCACCAGGGGCCCCTAAGCCGGACGGTAGGAAATCATATCAATGGCAGACTGCCACTAACCCGTCGGTATTCGCGAAATTGAGTGATCCACCCCCCCAGGTATCTGTCCCGTTCATGTCGCCAGCAACAGCTTATCAGTGGTTTTATGATGGCTACCCTACATTTGGTGAGCACAAACAAGCTACCAATTTGCAATATGGACAGTGTCCTAATAACATGATGGGCCATTTTGCCATCCGAACAGTCAGTGAATCTACTACCGGGAAAAACGTCCACGTTCGGGTGTACATGAGAATTAAGCACGTGAGAGCTTGGGTACCTAGACCCCTTCGATCCCAAGCTTATATGGTCAAGAACTATCCGACATACAGTCAAACAATAACTAACACTGCAACTGACCGTGCAAGCATAACCACTACAGATTATGAAGGCGGGGTACCAGCAAACCCACAAAGGACATCT

>MK167122.1

AATGATCCCATTACAAATGCAGTGGAAAGCGCTGTGAGCGCGCTTGCTGATACCACAATATCCCGGGTGACCGCAGCCAACACTGCAGCTAGCACCCACTCCCTGGGAACAGGGCGTGTACCAGCATTACAAGCTGCAGAAACGGGAGCAAGCTCTAATGCTAGTGATGAGAATCTTATTGAGACCCGCTGCGTGATGAATCGAAACGGGGTCAATGAGGCGAGTGTGGAGCACTTTTACTCTCGTGCAGGGCTGGTAGGAGTTGTGGAGGTAAAGGACTCAGGCACCAGCCTGGATGGGTACACAGTTTGGCCTGTAGATGTAATGGGCTTCGTGCAACAGCGGCGCAAGCTAGAGCTGTCAACATACATGCGCTTCGATGCCGAGTTCACCTTTGTATCCAACCTCAGTGACAGCACGACGCCCGGGATGCTGCTACAGTATATGTATGTACCACCAGGGGCCCCTAAGCCGGATAGCAGGAAATCATACCAATGGCAGACTGCTACTAACCCGTCGGTATTCGCAAAATTGAGTGATCCACCCCCTCAGGTGTCTGTCCCGTTCATGTCGCCAGCAACAGCTTATCAGTGGTTTTATGATGGTTATCCTACATTTGGTGAGCACAAACAGGCTACCAATTTGCAATATGGGCAGTGCCCTAACAATATGATGGGCCATTTTGCTATCCGAACAGTTAGTGAATCCACCACCGGAAAAAACGTCCACGTCCGGGTGTACATGAGAATTAAGCACGTGAGAGCTTGGGTACCTAGACCCCTTCGATCCCAGGCTTATATGGTCAAGAATTACCCGACATATAGCCAAACAATAACTAACACTGCAACTGACCGTGCAAGCATAACCACCACGGATTATGAAGGTGGGGTGCCAGCAAACCCACAAAGGACGTCT

>MK252982.1

AACGATCCCATTACAAATGCAGTGGAAAGCGCTGTGAGCGCGCTTGCTGACACCACAATATCCCGGGTGACCGCAGCCAACACTGCAGTTAGCACCCACTCCCTGGGAACAGGGCGTGTACCAGCATTGCAAGCTGCAGAAACGGGAGCAAGCTCTAATGCTAGTGATGAGAACCTTATTGAGACCCGCTGCGTGATGAATCGAAACGGGGTTAATGAGGCGAGTGTGGAACACTTTTACTCTCGTGCAGGGCTGGTAGGAGTTGTGGAGGTGAAGGACTCAGGCACCAGCCTGGATGGGTACACAGTTTGGCCTGTAGATGTAATGGGCTTCGTGCAACAGCGGCGCAAACTAGAGCTGTCAACATACATGCGCTTCGATGCCGAGTTCACCTTTGTATCCAACCTCAGTGACAGCACGACGCCCGGGATGCTGCTACAGTATATGTATGTACCACCAGGGGCCCCTAAGCCGGATAGCAGGAAATCATACCAATGGCAGACTGCTACTAACCCGTCGGTATTCGCAAAATTGAGTGATCCACCCCCCCAGGTGTCTGTCCCGTTTATGTCGCCAGCAACAGCTTATCAGTGGTTTTATGATGGTTACCCTACATTTGGTGAACACAAACAGGCTACCAATTTGCAATATGGGCAGTGCCCTAACAACATGATGGGCCATTTTGCTATCCGAACAGTTAGTGAATCTACCACCGGAAAAAACGTCCACGTCCGGGTGTACATGAGAATTAAGCACGTGAGAGCTTGGGTACCTAGACCCCTTCGATCCCAAGCTTATATGGTCAAGAATTACCCGACATATAGCCAAACAATAACTAACACTGCAACTGACCGTGCAAGCATAACCACCACGGATTATGAAGGTGGGGTGCCAGCAAACCCACAAAGGACATCT

>MK252991.1

AACGATCCCATTACAAATGCAGTGGAAAGCGCTGTGAGCGCGCTTGCTGACACTACAATATCCCGGGTGACCGCAGCCAATACTGCAGCTAGCACCCACTCCCTGGGAACAGGGCGTGTACCAGCATTACAAGCTGCAGAAACGGGAGCAAGCTCTAATGCTAGTGATGAAAACCTTATTGAGACCCGCTGCGTGATGAATCGAAACGGGGTTAATGAGGCGAGTGTGGAGCACTTTTACTCTCGTGCAGGGCTGGTAGGAGTTGTGGAGGTGAAGGACTCAGGCACCAGCCTGGATGGGTACACAGTTTGGCCTGTAGATGTAATGGGCTTCGTGCAACAGCGGCGCAAACTAGAGCTGTCAACATACATGCGCTTCGATGCCGAGTTCACCTTTGTATCCAACCTCAGTGACAGCACGACGCCCGGGATGCTGCTACAGTATATGTATGTACCACCAGGGGCCCCTAAGCCGGATGGCAGGAAATCATACCAATGGCAGACTGCTACTAACCCGTCGGTATTCGCAAAATTGAGTGATCCACCCCCTCAGGTGTCTGTCCCGTTCATGTCGCCAGCAACAGCTTATCAGTGGTTTTATGATGGTTACCCTACATTTGGTGAGCACAAACAGGCTACCAATTTGCAATATGGGCAGTGCCCTAACAATATGATGGGCCATTTTGCTATCCGAACAGTTAGTGAATCTACCACCGGAAAAAACGTCCACGTCCGGGTGTACATGAGAATTAAGCACGTGAGAGCTTGGGTACCTAGACCCCTTCGATCCCAGGCTTATATGGTCAAGAATTACCCGACATATAGCCAAACAATAACTAACACTGCAACTGACCGTGCAAGCATAACCACCACGGATTATGAAGGTGGGGTGCCAGCAAACCCACAAAGGACATCT

>MK357074.1

AATGATCCCATTACAAATGCAGTAGAAAGCGCTGTGAGTGCGCTTGCTGACACCACAATATCCCGGGTGACCGCAGCCAACACTGCAGCTAGCACCCACTCCCTGGGAACAGGGCGTGTACCAGCATTGCAAGCCGCAGAAACGGGAGCAAGCTCTAATGCCAGTGATGAGAACCTTATTGAGACTCGCTGTGTGATGAATCGAAACGGGGTTAATGAGGCGAGTGTAGAACACTTTTACTCTCGTGCAGGGCTGGTAGGAGTTGTAGAGGTGAAGGACTCGGGCACTAGCTTGGATGGGTACACAGTTTGGCCCATAGATGTGATGGGTTTCGTGCAACAGCGGCGCAAGCTAGAGTTGTCAACATACATGCGCTTTGATGCCGAGTTCACTTTTGTATCCAACCTCAATGACAGCACGACGCCCGGGATGTTGCTGCAGTACATGTATGTGCCACCAGGGGCCCCTAAGCCAGATAGCAGGAAGTCATATCAATGGCAGACTGCTACTAACCCATCGGTGTTCGCAAAATTGAGTGATCCACCCCCTCAGGTATCTGTCCCGTTCATGTCGCCAGCAACGGCTTATCAGTGGTTTTATGATGGTTACCCTACATTTGGTGAACATAAACAAGCTACCAATTTGCAATATGGGCAGTGTCCTAACAACATGATGGGCCATTTTGCTATCCGAACAGTCAGTGAATCCACCACCGGGAAAAACGTCCACGTTCGGGTATACATGAGAATTAAGCACGTGAGAGCTTGGGTACCTAGACCCCTTCGATCCCAAGCTTACATGGTCAAGAACTATCCGACATACAGCCAAACAATAACCAACACTGCAGCTGACCGTGCAAGCATAACCACCACGGATTATGAAGGCGGAGTACCAGCAAACCCACAGAGGACATCT

>MK652140.1

AACGATCCCATTACAAATGCAGTGGAAAGCGCTGTGAGCGCGCTTACTGACACCACAATATCCCGGGTGACCGCAGCCAACACTGCAGCTAGCACCCACTCCCTGGGAACAGGGCGTGTACCAGCATTACAAGCTGCAGAAACGGGAGCAAGCTCTAATGCTAGTGATGAGAACCTTATTGAGACCCGCTGTGTGATGAATCGAAACGGGGTTAATGAGGCAAGTGTGGAGCACTTTTACTCTCGTGCAGGGCTGGTAGGAGTTGTGGAGGTGAAGGACTCAGGCACCAGCCTGGATGGGTACACAGTTTGGCCTGTAGATGTAATGGGCTTCGTGCAACAGCGGCGCAAATTAGAGCTGTCAACATATATGCGCTTCGATGCTGAGTTCACCTTTGTATCCAATCTCAGTGACAGCACGACGCCCGGGATGCTGCTACAGTATATGTATGTACCACCAGGGGCCCCTAAGCCGGATAGCAGGAAATCATACCAATGGCAGACTGCTACTAACCCGTCGGTATTCGCAAAATTGAGTGATCCACCCCCTCAGGTGTCTGTCCCGTTCATGTCGCCAGCAACAGCTTATCAGTGGTTTTATGATGGCTACCCTACATTTGGTGAGCACAAACAGGCTACCAATTTGCAATATGGGCAGTGCCCTAACAATATGATGGGCCATTTTGCTATCCGAACAGTTAGTGAATCTACCACCGGAAAAAACGTTCACGTCCGGGTGTACATGAGAATTAAGCACGTGAGAGCTTGGGTACCTAGACCCCTTCGATCCCAGGCTTATATGGTCAAGAATTACCCAACATATAGCCAAACAATAACTAACACTGCAACTGACCGTGCAAGCATAACCACCACGGATTATGAAGGTGGGGTGCCAGCAAACCCACAAAGGACATCT

>MN336288.1

AATGATCCCATTACAAATGCAGTGGAAAGCGCTGTGAGCGCGCTTGCTGACACCACAATATCCCGGGTGACCGCAGCCAACACTGCAGCTAGCACCCACTCCCTGGGAACAGGGCGTGTACCAGCATTGCAAGCCGCAGAAACGGGAGCAAGCTCTAATGCTAGTGATGAGAACCTTATTGAGACTCGCTGTGTGATGAATCGAAACGGGGTTAATGAGGCGAGTGTGGAACACTTTTACTCTCGTGCAGGGCTGGTAGGAGTTGTGGAGGTGAAGGACTCGGGCACTAGCCTGGATGGGTACACAGTTTGGCCCATAGATGTGATGGGCTTCGTGCAACAGCGGCGCAAGCTAGAGCTGTCAACATACATGCGCTTTGATGCCGAGTTCACTTTTGTGTCCAACCTCAATGATAGCACGACGCCCGGGATGCTGCTGCAGTATATGTATGTACCACCAGGGGCCCCTAAGCCGGATAGCAGGAAATCATATCAATGGCAGACCGCTACTAACCCGTCGGTGTTCGCAAAATTGAGTGATCCACCCCCCCAGGTATCTGTTCCGTTCATGTCGCCAGCAACAGCTTATCAGTGGTTTTATGATGGTTACCCTACATTTGGTGAGCACAAACAAGCTACTAATTTGCAATATGGGCAGTGTCCTAATAACATGATGGGCCATTTTGCCATCCGAACAGTCAGTGAATCTACCACCGGGAAAAACGTCCACGTTCGGGTGTACATGAGAATTAAGCACGTGAAAGCTTGGGTACCTAGACCCCTTCGATCCCAAGCTTATATGGTCAAGAACTACCCCACATACAGCCAAACAATAACTAACACTGCAACTGACCGTGCAAGCATAACCACCACGGATTATGAAGGCGGGATACCAGCAAACCCACAAAGGACATCT

>MN336309.1

AATGATCCCATTACAAATGCAGTGGAAAGCGCTGTGAGTGCGCTTGCTGACACCACAATATCCCGGGTAACCGCAGCCAACACTGCAGCTAGCACCCACTCCCTGGGAACAGGGCGTGTACCAGCATTGCAAGCCGCAGAAACGGGAGCAAGCTCTAATGCTAGTGATGAGAACCTTATTGAGACCCGCTGTGTGATGAATCGAAACGGGGTCAATGAGGCGAGTGTGGAACACTTTTACTCTCGAGCAGGACTGGTAGGAGTTGTGGAGGTGAAGGACTCGGGCACTAGCCTGGATGGGTACACAGTTTGGCCCATAGATGTGATGGGCTTCGTGCAACAGCGGCGCAAGCTAGAGCTGTCAACATACATGCGCTTTGATGCCGAGTTCACTTTTGTGTCCAACCTCAATGACAGCACGACACCCGGGATGCTGCTGCAGTATATGTATGTACCACCAGGGGCCCCTAAGCCGGATAGCAGGAAATCATATCAATGGCAGACTGCTACTAACCCGTCGGTATTCGCAAAATTGAGTGATCCACCCCCCCAGGTATCTGTTCCGTTCATGTCGCCAGCAACAGCTTATCAGTGGTTTTATGATGGTTACCCTACATTTGGTGAGCACAAACAAGCTACCAACTTGCAATATGGGCAGTGTCCTAATAACATGATGGGCCATTTTGCCATCCGAACAGTCAGTGAATCTACCACCGGGAAAAATGTCCACGTTCGGGTGTACATGAGAATTAAGCACGTGAGAGCTTGGGTACCTAGACCCCTTCGATCCCAAGCTTATATGGTCAAGAACTACCCGACATACAGCCAAACAATAACTAACACTGCAACTGACCGCGCAAGCATAACCACCACGGATTATGAAGGCGGAGTGCCAGCAAACCCACAAAGGACATCT

>MN336316.1

AATGATCCCATCACAAATGCAGTGGAAAGCGCTGTGAGCGCGCTTGCTGACACCACAATATCCCGGGTGACTGCAGCCAGTACCGCAGCTAGCACCCACTCTCTGGGAACAGGGCGTGTACCAGCATTGCAAGCCGCAGAAACGGGAGCAAGCTCTAATGCTAGTGATGAGAACCTTATTGAGACTCGCTGTGTGATGAATCGAAACGGGGTTAATGAGGCGAGTGTGGAACACTTTTACTCTCGTGCAGGGCTAGTAGGAGTTGTGGAGGTGAAGGACTCGGGTACTAGCTTGGATGGGTACACAGTTTGGCCCATAGATGTGATGGGCTTTGTGCAACAGCGGCGCAAGCTAGAGCTGTCAACATACATGCGCTTTGATGCCGAGTTCACTTTTGTGTCCAACCTCAATGACAGCACGACGCCTGGGATGCTGCTACAGTACATGTATGTACCACCAGGGGCCCCTAAGCCGGATAGCAGGAAATCATATCAATGGCAGACTGCTACTAACCCGTCAGTATTTGCAAAATTGAGTGATCCACCCCCCCAGGTATCTGTTCCGTTCATGTCACCAGCAACAGCTTATCAGTGGTTTTATGATGGTTACCCTACATTTGGTGAGCACAAACAAGCTACCAATTTGCAATATGGGCAGTGTCCTAATAACATGATGGGCCATTTTGCCATCCGAACAGTCAGTGAATCTACCACCGGGAAAAACGTCCACGTTCGGGTGTACATGAGAATTAAGCACGTGAGAGCTTGGGTACCTAGACCCCTTCGATCCCAAGCCTATATGGTTAAGAACTACCCGACATACAGCCAAACAATAACTAACACTGCAACTGACCGTGCAAGCATAACCACCACGGATTATGAAGGTGGGGTACCAGCAAACCCACAAAGGACATCT

>MN341008.1

AATGATCCCATCACAAATGCAGTAGAAAGCGCTGTGAGTGCGCTTGCTGACACCACAATATCCCGAGTGACCGCAACCAACACTGCAGCTAGCACCCACTCCCTGGGAACAGGGCGTGTACCAGCATTGCAAGCCGCAGAAACGGGAGCAAGCTCTAATGCCAGTGATGAGAACCTTATTGAGACTCGCTGTGTGATGAATCGAAACGGGGTTAATGAGGCGAGTGTAGAACACTTTTACTCTCGTGCAGGACTGGTAGGAGTTGTGGAGGTGAAGGACTCGGGCACTAGCTTGGATGGGTACACAGTTTGGCCCATAGATGTGATGGGCTTCGTGCAACAGCGGCGCAAGCTAGAGTTATCAACATACATGCGCTTTGATGCCGAGTTCACTTTTGTATCCAACCTCAATGACAGCACGACGCCCGGGATGTTGCTGCAGTACATGTATGTGCCACCAGGGGCCCCTAAGCCAGATAGCAGGAAGTCATATCAATGGCAGACTGCTACTAACCCATCGGTATTCGCAAAATTGAGTGATCCACCCCCTCAGGTATCTGTCCCGTTCATGTCGCCAGCAACGGCTTATCAGTGGTTTTATGATGGTTACCCTACATTTGGTGAACACAAACAAGCTACCAATTTGCAATATGGGCAGTGTCCTAACAACATGATGGGCCATTTTGCTATCCGAACAGTCAGTGAATCCACCACCGGGAAAAACGTCCACGTTCGGGTATACATGAGAATTAAGCACGTGAGAGCTTGGGTGCCTAGGCCCCTTCGATCCCAAGCTTACATGGTCAAAAACTATCCGACGTACAGCCAAACAATAACCAACACTGCAGCTGACCGTGCAAGCATAACCACCACGGATTATGAAGGCGGGGTACCAGCAAACCCACAGAGGACATCT

>MN689954.1

AATGATCCCATTACAAATGCAGTGGAAAGCGCTGTGAGCGCGCTTGCCGACACCACAATATCCCGGGTGACCGCAGCCAACACTGCAGCTAGCACCCACTCCCTGGGAACAGGGCGTGTACCAGCACTGCAAGCCGCAGAAACGGGAGCAAGCTCTAATGCCAGTGATGAGAGCCTTATTGAGACCCGCTGTGTGATGAATCGAAACGGGGTTAATGAGGCGAGTGTGGAACACTTTTACTCTCGTGCAGGGCTGGTAGGAGTTGTGGAGGTGAAGGACTCGGGCACTAGCCTGGATGGGTACACAGTTTGGCCCATAGATGTGATGGGCTTCGTGCAACAGCGGCGCAAGCTAGAGTTGTCAACATACATGCGCTTTGATGCCGAGTTCACTTTTGTGTCCAACCTCAATGATAGCACGGCGCCCGGGATGCTGCTGCAGTATATGTATGTGCCACCAGGGGCCCCTAAGCCAGACAGCAGGAAATCATACCAATGGCAGACTGCTACTAACCCGTCGATATTCGCAAAGTTGAGTGATCCACCCCCCCAGGTGTCTGTCCCGTTCATGTCGCCAGCAACGGCTTATCAGTGGTTTTATGATGGTTACCCTACATTTGGTGAACACAAACAAGCTACCAATTTGCAATATGGGCAGTGTCCTAATAACATGATGGGCCATTTTGCTATCCGAACAGTCAGTGAATCTACCACCGGGAAAAACGTCCACGTTCGGGTGTACATGAGAATTAAGCACGTGAGAGCTTGGGTACCTAGACCCCTTCGATCCCAAGCTTATATGGTCAAGAACTACCCGACATACAGCCAAACAATAACTAACACTGCAGCTGACCGTGCAAGCATAACCACCACGGATTATGAAGGTGGGGTACCAGCAAACCCACAGAGGACATCT
