# Supplementary material for: One-Step Reverse-Transcription Recombinase Polymerase Amplification Using Lateral Flow Strips for the Detection of Coxsackievirus A6
Source: Front Microbiol. 2021 Feb 4;12:629533. doi: 10.3389/fmicb.2021.629533 (PMC7889601; doi:10.3389/fmicb.2021.629533)
Supplement: Supplementary Material 2 — Detection of CVA-6 using the enclosed disposable device. P, positive control; N, negative control. [file Data_Sheet_2.docx]

**Supplementary file 2**

Detection of CVA-6 using the enclosed disposable device. P: positive control; N: negative control.
